# Vegetative desiccation tolerance in the resurrection plant *Xerophyta humilis* has not evolved through reactivation of the seed canonical LAFL regulatory network

**DOI:** 10.1111/tpj.14596

**Published:** 2019-12-10

**Authors:** Rafe Lyall, Stephen A. Schlebusch, Jessica Proctor, Mayur Prag, Steven G. Hussey, Robert A. Ingle, Nicola Illing

**Affiliations:** ^1^ Department of Molecular and Cell Biology University of Cape Town Rondebosch 7701 South Africa; ^2^ Department of Biochemistry, Genetics and Microbiology, Forestry and Agricultural Biotechnology Institute (FABI) University of Pretoria Pretoria 0002 South Africa

**Keywords:** desiccation tolerance, resurrection plant, seed maturation, LAFL, ABI3, *Xerophyta humilis*, *Xerophyta viscosa*

## Abstract

It has been hypothesised that vegetative desiccation tolerance in resurrection plants evolved via reactivation of the canonical LAFL (i.e. *LEC1*, *ABI3*, *FUS3* and *LEC2*) transcription factor (TF) network that activates the expression of genes during the maturation of orthodox seeds leading to desiccation tolerance of the plant embryo in most angiosperms. There is little direct evidence to support this, however, and the transcriptional changes that occur during seed maturation in resurrection plants have not previously been studied. Here we performed *de novo* transcriptome assembly for *Xerophyta humilis*, and analysed gene expression during seed maturation and vegetative desiccation. Our results indicate that differential expression of a set of 4205 genes is common to maturing seeds and desiccating leaves. This shared set of genes is enriched for gene ontology terms related to abiotic stress, including water stress and abscisic acid signalling, and includes many genes that are seed‐specific in *Arabidopsis thaliana* and targets of ABI3. However, while we observed upregulation of orthologues of the canonical LAFL TFs and *ABI5* during seed maturation, similar to what is seen in *A. thaliana*, this did not occur during desiccation of leaf tissue. Thus, reactivation of components of the seed desiccation program in *X. humilis* vegetative tissues likely involves alternative transcriptional regulators.

## Significance Statement

A key question is whether vegetative desiccation tolerance in resurrection plants evolved through the ectopic expression of seed maturation transcription factors (TFs) in leaves. While the full‐length ABI3 and ABI5 master seed TFs are expressed at high levels in the maturing seed of *Xerophyta humilis*, they are silent in desiccating leaves. Desiccation tolerance in Xerophyta leaves must instead have evolved by rewiring of the vegetative stress response pathway to activate seed genes.

## Introduction

Plant adaptations to water stress form a spectrum that includes drought avoidance, drought tolerance and, at the extreme end of the scale, vegetative desiccation tolerance (VDT; Alpert, 2005). VDT is defined as survival after loss of > 95% of cellular water (Gaff and Oliver, 2013). It is very common in early diverging plants, such as mosses, liverworts and hornworts, where it is believed to have been the ancestral state, and vital for the colonisation of land by the ancestors of terrestrial plants (Oliver *et al.*, 2000). With the evolution and improvement of the plant vascular system, VDT was gradually lost from tracheophyte lineages. However, VDT re‐evolved independently in monocot and eudicot plant families, and is known to occur in over 135 taxonomically diverse species across 44 genera. These plant species are commonly referred to as ‘resurrection plants’, and are found across central and southern Africa, Europe, Asia, South America and Australia (Behnke *et al.*, 2013; Rakić *et al.*, 2014; Xiao *et al.*, 2015). A key question is how VDT evolved multiple times in these diverse plant lineages and whether it can be engineered into crop plants.

Although desiccation tolerance (DT) was lost in the vegetative tissues of vascular plants, the processes and networks involved in primitive VDT were not abandoned completely. Instead, these mechanisms were co‐opted by early vascular plants for abiotic stress responses and the protection of their reproductive propagules (Oliver *et al.*, 2000). Over 95% of angiosperms and gymnosperms produce orthodox seeds that contain DT embryos (Gaff and Oliver, 2013). Although the leaves and roots of most angiosperms do not exhibit VDT, there is nonetheless a narrow developmental window post‐germination in which seedlings are able to re‐establish DT in response to stress (Buitink *et al.*, 2006; Maia *et al.*, 2011; Lyall *et al.*, 2014). This phenomenon has been observed in the seedlings of several species, including *Cucumis sativus*, *Impatiens walleriana* and *Triticum aestivum* (Bruggink and van der Toorn, 1995, 1997; Farrant *et al.*, 2004), and has been investigated in both *Arabidopsis thaliana* and *Medicago truncatula* (Terrasson *et al.*, 2013; Maia, 2014; Dekkers *et al.*, 2015).

In orthodox seeds, the onset of embryonic DT occurs during the late maturation stage of embryogenesis as a pre‐programmed developmental transition (Angelovici *et al.*, 2010). Maturation and maturation drying are characterised by the accumulation of storage compounds (sugars, triglycerides and seed storage proteins), late embryogenesis abundant proteins (LEAs) and anti‐oxidants, which act as both osmoprotectants during desiccation and nutrient reserves during germination (Bewley and Black, 1994; Vicente‐Carbajosa and Carbonero, 2005; Dekkers *et al.*, 2015; Leprince *et al.*, 2017). Notably, many of the genes involved in seed maturation are also upregulated during the re‐acquisition of DT in germinating seedlings and in desiccating leaves of resurrection plants (Illing *et al.*, 2005; Buitink *et al.*, 2006; Terrasson *et al.*, 2013; Maia, 2014), but not in the leaves of desiccation‐sensitive plant species. It has been proposed that the similarity in protection mechanisms of seed maturation, seedling DT and VDT is evidence for a common regulatory mechanism between these processes, possibly via co‐option of the seed maturation gene networks during vegetative stress in resurrection plants (Oliver *et al.*, 2000; Illing *et al.*, 2005; VanBuren, 2017; Costa, Cooper, *et al.*, 2017).

In *A. thaliana*, the canonical LAFL network of seed master transcriptional regulators *LEAFY COTYLEDON 1* (*LEC1*), *ABSCISIC ACID INSENSITIVE 3* (*ABI3*), *FUSCA 3* (*FUS3*) and *LEAFY COTYLEDON 2* (*LEC2*) (Santos‐Mendoza *et al.*, 2008; North *et al.*, 2010) activates the expression of seed maturation genes in response developmental cues and abscisic acid (ABA) signalling. The B3 DNA binding domain transcription factor (TF) ABI3 plays a central role in the maturation response and has functional orthologues in many angiosperm species (Hattori *et al.*, 1994; Shiota *et al.*, 1998; Suzuki *et al.*, 2001; Footitt *et al.*, 2003; Carbonero *et al.*, 2016). Seeds of *abi3* mutants are desiccation‐sensitive in *A. thaliana* and *M. truncatula* (Nambara *et al.*, 1995; Terrasson *et al.*, 2013). *ABI3* orthologues have also been identified in basal land plants, including *Physcomitrella patens* (Khandelwal *et al*., 2010), and *abi3* deletion mutants of *P. patens* are unable to recover from desiccation, suggesting that this TF is a conserved and ancestral regulator of DT (Marella *et al.*, 2006; Khandelwal *et al*., 2010; Komatsu *et al.*, 2013).

The ABI3 protein is comprised of several conserved structural domains: the acidic regions A1 and A2, separated by a Pro‐, Ser‐ and Thr‐rich (PST) region, and the basic domains B1, B2 and B3 (Giraudat *et al.*, 1992). The A1 and A2 domains are transcriptional activators (McCarty *et al.*, 1991; Gagete *et al.*, 2009), while the B3 DNA‐binding domain binds the RY motif (CATGCA) commonly found in the promoters of seed maturation genes (Suzuki *et al.*, 1997; Sasnauskas *et al.*, 2018). The B2 domain contains a nuclear localisation signal and appears to have transactivation capability (Marella *et al.*, 2006), but can also mediate gene expression indirectly via the ABA response element (ABRE), most likely through protein−protein interactions (Ezcurra *et al.*, 2000). The B1 domain can interact with other proteins, especially those of the bZIP TF family and in particular ABI5 (Nakamura *et al.*, 2001), which is known to bind to ABREs. ABI5, a member of the ABA‐responsive (group A) bZIP family of TFs, acts downstream of the canonical LAFL network and, like ABI3, is essential for seed desiccation (Santos‐Mendoza *et al.*, 2008). Furthermore, both ABI3 and ABI5 are important for the re‐acquisition of DT in germinating seedlings in response to ABA signalling (Lopez‐Molina *et al.*, 2001; Terrasson *et al.*, 2013).

The genomes of several resurrection plant species have been sequenced in recent years, including the monocots *Oropetium thomaeum* and *Xerophyta viscosa*, and the eudicots *Boea hygrometrica* and *Lindernia brevidens* (VanBuren *et al.*, 2015, 2018; Wen *et al.*, 2015; Costa, Ligterink, *et al.*, 2017). RNA‐Seq analysis of the transcriptional changes occurring during desiccation of leaf tissue has also been performed for a number of additional species, including *Craterostigma plantagineum* (Rodriguez *et al.*, 2010), *Sporobolus stapfianus* (Yobi *et al.*, 2017), *B. hygrometrica* (Xiao *et al.*, 2015) and *Haberlea rhodopensis* (Gechev *et al.*, 2013). While these studies have demonstrated that many genes induced during seed maturation are also upregulated in desiccating leaves of resurrection plants, the question as to how these seed maturation genes are reactivated in desiccating leaves remains unanswered. It has been speculated that members of the canonical LAFL network perform this role (Costa, Cooper, *et al.*, 2017; VanBuren, 2017), but there is little direct evidence to support this hypothesis. For example, although *X. viscosa* homologues of many of the *A. thaliana* genes directly regulated by ABI3 (the ABI3 regulon) were upregulated in both leaves and seedlings during desiccation or ABA treatment, transcript levels of two *X. viscosa ABI3* orthologues were not (Costa, Ligterink, *et al.*, 2017). Furthermore, no transcriptomic analysis of seed maturation in resurrection plants has been undertaken. Thus, it is not possible to know whether the transcriptional reprogramming that occurs during this developmental process is analogous to that occurring in *A. thaliana*, or whether the same TFs regulate genes important for both seed and vegetative DT in resurrection plants.

In this study we present an RNA‐Seq transcriptome assembly and analysis of gene expression during seed maturation and vegetative desiccation in the monocot resurrection plant *Xerophya humilis*. Our results suggest that the expression of seed‐specific genes during VDT is highly unlikely to result from simple reactivation of the canonical LAFL network or known seed regulatory TFs (such as *ABI5*). Instead, we hypothesise that in vegetative tissues the expression of the ABI3 regulon is activated by a desiccation‐responsive pathway, which may include members of the ABRE‐binding factor (ABF) family of TFs.

## Results

### Stages of development of *Xerophyta humilis* seeds

**Figure 1:**
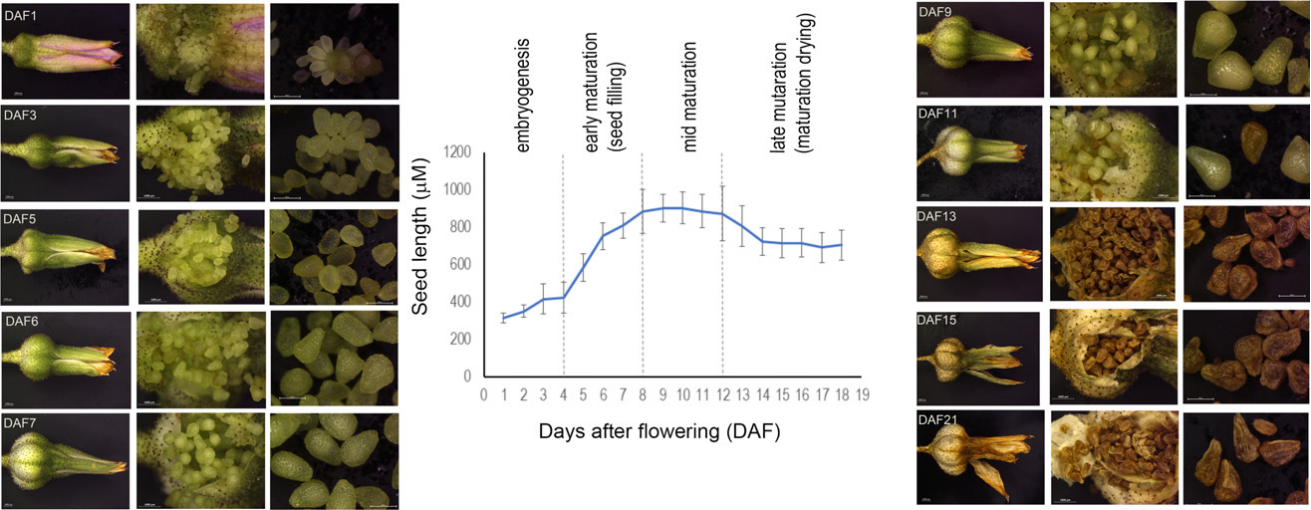
Overview of *Xerophyta humilis* seed development. Left and right: Images showing *X. humilis* flowers, seed pods and seeds at different days after fertilisation (DAF). Centre: *X. humilis* seed length as development progresses. Seeds show a rapid increase in size between DAF4 and DAF8, consistent with early maturation and seed filling. Seed length is stable during mid maturation (DAF8−DAF12) and drops slightly as seeds lose water during late maturation (DAF12+). For this study, seeds were sampled at DAF6 (early maturation), DAF11 (mid maturation) and DAF17 (late maturation/dry). A range of 34–161 seeds were measured from across three seed pods for each DAF; error bars are standard deviation.

Seed development in *X. humilis* takes 2–3 weeks from fertilisation to dry seed. The early embryogenesis stages, characterised by a slow increase in seed size, occurred between 1 and 4 days after fertilisation (DAF1−DAF4; Figure 1). Seed size increased rapidly thereafter (DAF5−DAF8), which likely represents the switch from embryogenesis to seed filling and early maturation, as observed in other angiosperms (Dam *et al.*, 2009; Garg *et al.*, 2017). Over the next few days, during the mid‐maturation stage (DAF9−DAF12), the size of the seeds did not change substantially, and they lost their green colouration. Late maturation occurred from DAF13 to DAF17 when seeds shrunk and turned brown. Dry seeds (DAF14+) were wrinkled and spilled easily from the seed pod (Figure 1). For this study, seed developmental stages representing early seed maturation (DAF6; small, green seeds), mature green (DAF11; large, green seeds) and mature dry seeds (DAF17; shrunken, brown seeds with little moisture) were selected for RNA‐Seq.

### Assembly and annotation of the *Xerophyta humilis* seed and leaf transcriptome

Independent RNA‐Seq experiments were performed on RNA extracted from *X. humilis* seeds during three stages of seed maturation (DAF6, DAF11 and DAF17, totalling 9 libraries and 193 million read pairs) and from leaves harvested at five relative water contents (RWCs) during a course of vegetative desiccation (100%, 80%, 60%, 40% and 5% RWC, totalling 15 libraries and 350 million read pairs).

After trimming, filtering and error correction, the reads from both experiments were combined for *de novo* transcriptome assembly. Over 500 000 contigs (350 000 Trinity genes) were assembled in the raw assembly, with an N50 of 1198 bp and an average read mapping rate of 81% (seed libraries) and 77% (leaf libraries). RapClust was used to remove low‐evidence contigs and cluster transcripts based on fragment equivalence classes computed by Salmon (Patro *et al.*, 2017). We leveraged a draft *X. humilis* genome (Schlebusch, 2019) to collapse these groups by combining transcript clusters that mapped to the same genomic locus. The resultant RapClust assembly consisted of 120 158 genes (where each ‘gene’ consists of one or more alternatively spliced transcripts). Putative open reading frames (ORFs) for each gene were determined using TransDecoder, which identified 25 391 genes containing a complete ORF and a further 35 868 that contained a partial ORF. Representative transcripts from each gene were defined based on length of the longest predicted complete ORF or, if lacking an ORF, the longest contig sequence.

TransRate and the Benchmarking Universal Single‐Copy Orthologs (BUSCO) programs were used to assess the quality and completeness of the *de novo* transcriptome assemblies, respectively. The TransRate scores of the raw assembly were low due to the very large number of low‐evidence contigs (assembled from background reads, and reads containing SNPs and errors). Both scores improved substantially when using the RapClust assembly, and the optimised score fell within the 25–50th percentile of the optimised scores calculated for 155 published assemblies by Smith‐Unna *et al. *(2016; Figure S1a). In order to assess the completeness and integrity of the assembly, the transcriptome was scanned for the presence of 1440 plant BUSCOs. Over 70% of BUSCOs were present as full‐length sequences in the RapClust assembly, with 90% of all BUSCOs present as complete or partial sequences (Figure S1b).

To predict potential gene identity and function, a representative transcript from each gene (encoding the longest ORF) was compared with protein sequences in the Swissprot and Uniref90 databases. In total, 40 567 (34%) transcripts had a significant blast match at an e‐value of < 1e^‐6^. Of these, the vast majority (80%) were against plant proteins (Figure S1c). The remaining top blast hits (20%) were against proteins of fungal origin, but < 0.5% of reads in each library mapped against these sequences suggesting that they likely originated from low‐level fungal contamination of the field‐collected plants. Annotations were assigned using Blast2GO, resulting in the successful annotation of 34 783 genes using the default evidence weightings, matching to 19 956 individual protein entries in the Swissprot and Uniref90 public databases.

### Comparison of gene expression during seed maturation and vegetative desiccation tolerance

**Figure 2:**
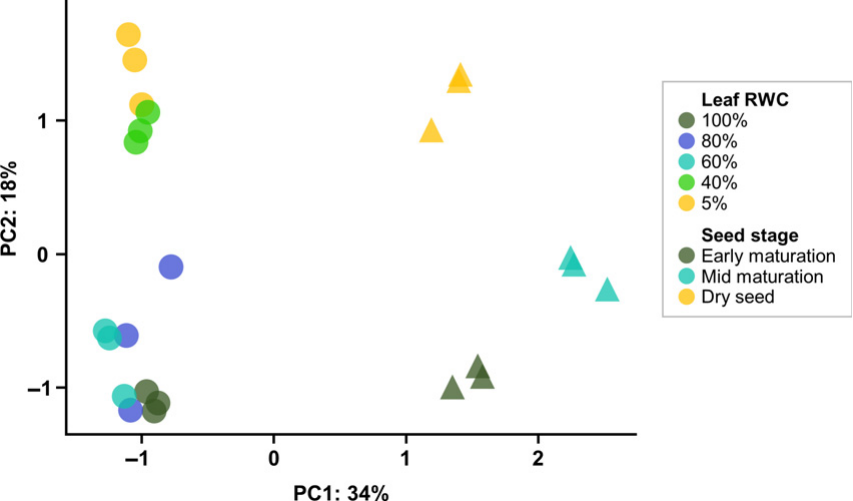
Principal component analysis (PCA) plot of seed and leaf RNA‐Seq libraries. Plots were generated using regularised log‐normalised read counts. PC1 separates samples on experiment (tissue type) and PC2 by water content.

Principal component analysis (PCA) of regularised log‐normalised read count data showed that the RNA‐Seq libraries separated primarily by experiment (tissue of origin) and secondarily by water content (Figure 2). In leaf tissues, there was a clear separation of samples at higher water contents (100%, 80% and 60% RWC) and samples during the later stages of desiccation (40% and 5% RWC), suggestive of a large shift in gene expression. Recently, it has been argued that a RWC of 40% represents the boundary between dehydration and desiccation, as defined by physiological and molecular changes observed across several resurrection plants (Zhang and Bartels, 2018).

**Figure 3:**
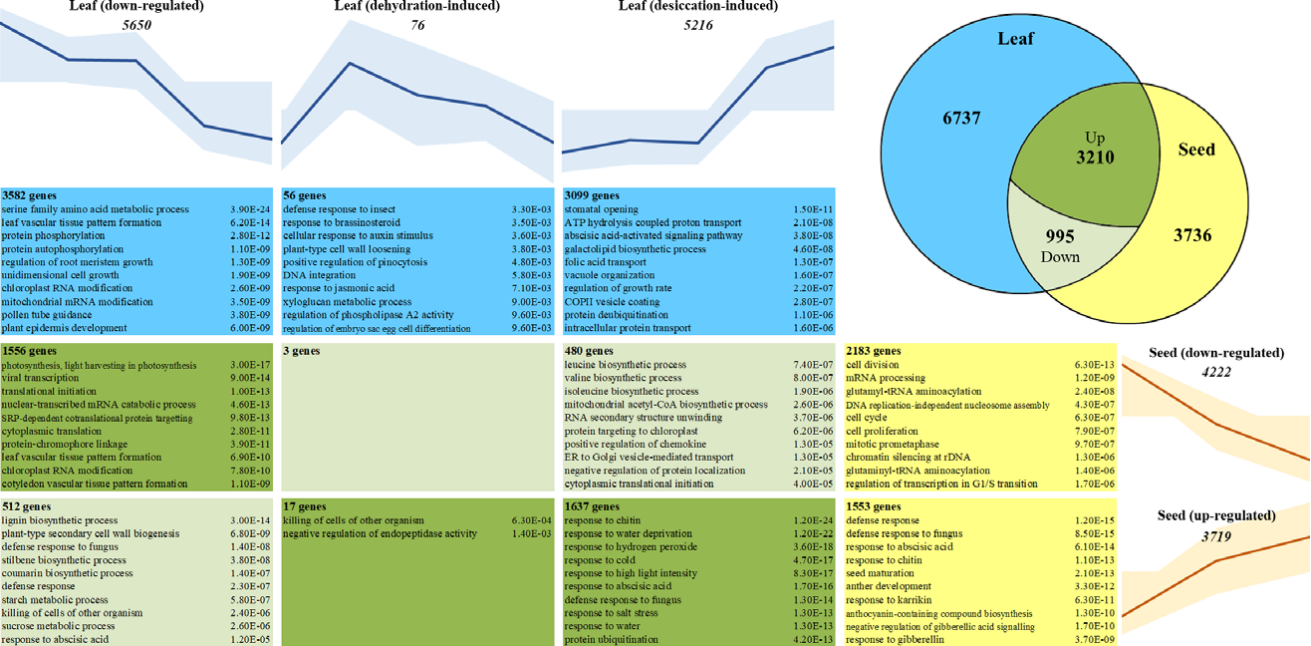
Number, overlap and gene ontology (GO) enrichment of genes differentially expressed (DE) during vegetative desiccation tolerance (VDT) and seed maturation. Genes only DE in leaf tissue (blue) are grouped based on expression pattern as downregulated (most highly expressed at 100% relative water content (RWC)), dehydration‐induced (most highly expressed at 80–60% RWC) or desiccation‐induced (most highly expressed at 40–5% RWC). Seed‐specific DE genes (yellow) are classified as downregulated (expressed at early maturation) or upregulated (most highly expressed mid−late maturation). Gene sets that are DE in both tissues are shown in green, with the darker shade indicating those that display the same direction of regulation during VDT and seed maturation. Expression profiles show the average scaled expression with standard deviation (shaded) for each gene in that cluster. The number of genes in each group is indicated, as are the 10 most significantly over‐represented GO terms in that set, in descending order of significance (*P* < 0.01).

The degree to which the transcriptional reprogramming occurring during seed maturation and VDT overlaps in resurrection plants is unknown. To address this, we identified genes that were differentially expressed (DE) during VDT and/or seed maturation, and then compared their expression profiles during the two processes. DE genes were grouped into simple expression clusters based on the point of maximal expression. In leaves this resulted in three groups: downregulated (maximal expression at 100% RWC); early dehydration‐induced (maximal expression at 80% or 60% RWC); and desiccation‐induced (maximal expression at 40% or 5% RWC) as defined by Zhang and Bartels (2018). For seed maturation, we used only two groups: downregulated genes (maximal expression during early maturation) and upregulated genes (genes with maximal expression at either mid or late maturation). The average expression profile for the genes in each group can be seen in Figure 3. To validate the RNA‐Seq data, we used quantitative polymerase chain reaction (qPCR) to independently analyse the expression of three genes that are upregulated during desiccation in leaf tissue according to our DeSEQ2 analysis, and observed good agreement between the expression values derived from the two methods (Figure S2).

A total of 14 678 *X. humilis* genes were DE, of which 6737 (46%) were DE in drying leaves only, 3736 (25%) in maturing seeds only, and 4205 (28%) during both processes (Figure 3). The number of genes that were specifically induced during the early dehydration phase in leaves (80% and 60% RWC) was very low (76 genes). Of the 4205 genes DE in both seed and leaf, 76% showed the same direction of DE (i.e. either upregulated in both tissues or downregulated in both; Figure 3). These results suggest that there is indeed a degree of overlap in the transcriptional reprogramming that occurs during VDT and seed maturation in *X. humilis.*

To identify the potential roles that might be played by these gene sets, we looked for enrichment of GO terms within them. Interestingly, GO terms associated with abiotic stress, including water stress, are not enriched in the leaf‐ or seed‐specific gene sets. Instead, most of the GO terms that might be expected to be associated with VDT were only associated with genes that were DE during both vegetative desiccation and seed maturation (Figure 3). For example, as a poikilochlorophyllous resurrection plant, *X. humilis* ceases photosynthesis and dismantles its thylakoid membranes during desiccation (Ingle *et al.*, 2008). GO terms related to photosynthesis and response to light were enriched only in the genes downregulated during both seed maturation and VDT. This suggests that many of the genes involved in photosynthetic and chloroplast structural changes during VDT may be shared with processes in the seed. Similarly, the 1637 genes that are upregulated during vegetative desiccation and seed maturation were enriched for a broad range of terms associated with response to stress, including response to water, water deprivation, cold, H_2_O_2_, high light, chitin and salt − terms that are absent from other gene sets (Figure 3).

### Homologues of *Arabidopsis thaliana* seed genes are upregulated during both seed maturation and vegetative desiccation tolerance in *Xerophyta humilis*

**Figure 4:**
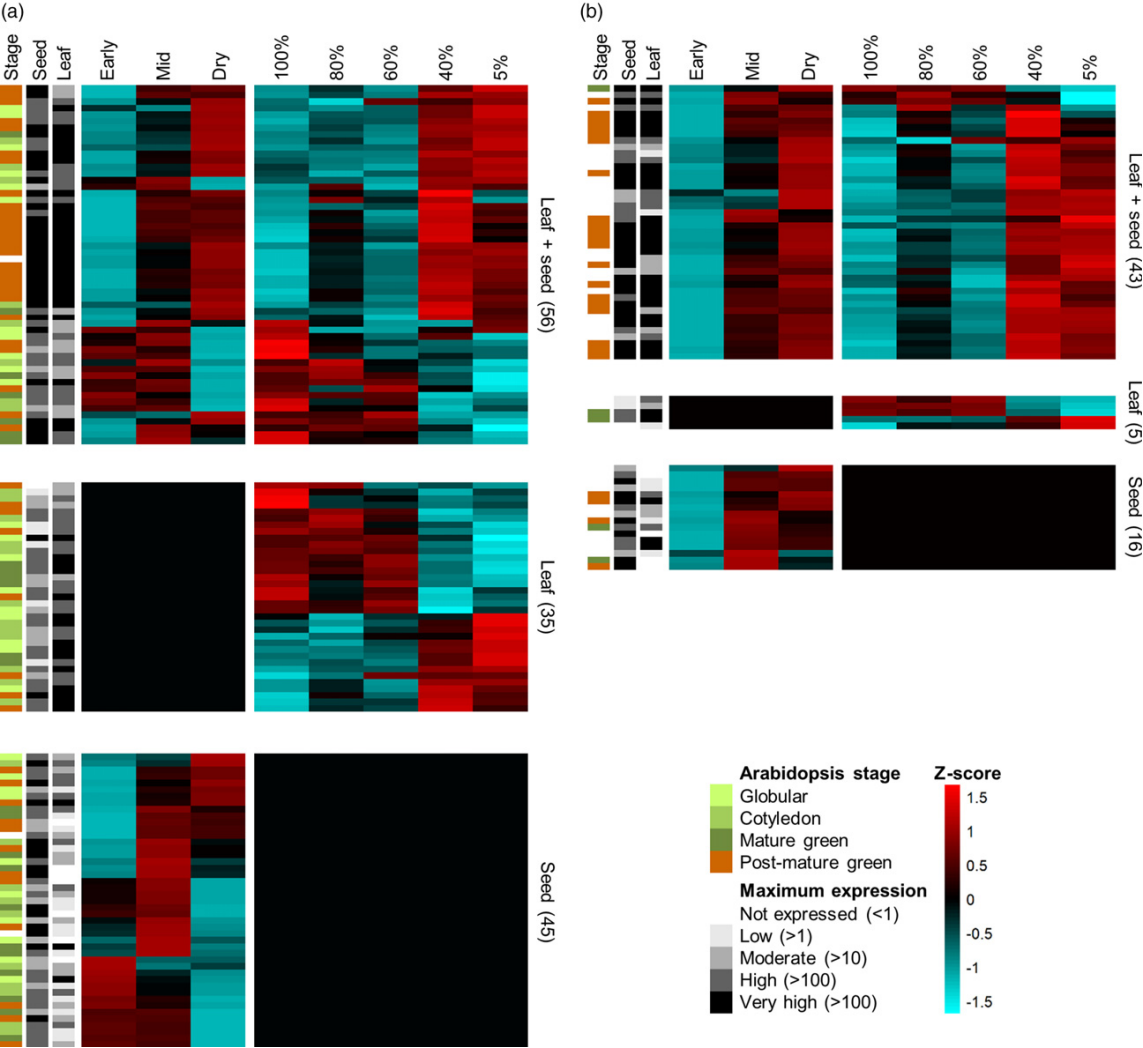
Expression patterns of *Xerophyta humilis* homologues of *Arabidopsis thaliana* seed‐specific and ABI3 regulon genes. Heatmaps profiling expression of *A. thaliana* seed‐specific genes (a) and 64 putative homologues of the ABI3 regulon in *X. humilis* (b) during seed maturation and vegetative desiccation tolerance (VDT). Genes that are not differentially expressed (DE) during either one of the processes have been given a scaled expression value (*z*‐score) of 0 in that tissue. Annotations on the left of both graphs represent whether the homologue in *A. thaliana* reached maximum expression during the globular (GLOB), cotyledon (COT), mature green (MG) or post‐mature green (PMG) stage of seed development in that species, based on data from Le *et al*. (2010). The read count at the maximum expression level during seed maturation (‘Seed’) and VDT (‘Leaf’) for each given gene is shown in a grey scale.

While both seed maturation and VDT in *X. humilis* involve the upregulation of a shared subset of genes, it is still unclear to what extent VDT‐associated genes in *X. humilis* are canonically seed‐specific in desiccation‐sensitive plants. We thus analysed the expression of the *X. humilis* homologues of a set of genes previously identified as seed‐specific in *A. thaliana*. Our *X. humilis* transcriptome contains 204 potential homologues to 152 of the 289 seed‐specific genes identified by Le *et al. *(2010), of which 136 (67%) were DE (Figure 4a; Data S1).

In general, genes that were DE in both tissues (56 in total) displayed the same expression profile (up in seed and leaf, or vice versa; Figure 4a). Additionally, in the leaf, there is again a clear switch in gene expression that occurs for these genes between 60% and 40% RWC. Thirty‐five *A. thaliana* seed‐specific genes were not identified as being DE during seed maturation in *X. humilis* despite showing a change in expression during VDT (Figure 4a). However, the majority of these genes reached peak expression early during *A. thaliana* seed development (globular, cotyledon or mature green stages) and, as our RNA‐Seq analysis was restricted to maturing seed tissues only, they may have been activated earlier in *X. humilis* seed development and not identified as DE in this study. Homologues of genes identified as being seed‐specific in Arabidopsis, but which were not DE in either *X. humilis* seeds or leaves, are listed in Data S1, together with their average expression. Absent from this list were genes linked to constitutive desiccation protection in some resurrection plants; for example, LEAs, dehydrins, antioxidant enzymes or transketolases (Oliver *et al.*, 2005; Dinakar and Bartels, 2013).

The 37 genes upregulated during both seed maturation and VDT (Figure 4a, upper segment) contained a significantly higher proportion of genes that were specific to the post‐mature green (PMG) stage of *A. thaliana* seed development (24/37 genes) compared with genes with other expression patterns (25/99 genes; *χ* ^2^ *P* = 0.0004). All these PMG‐associated genes were homologues of genes from the *A. thaliana* ABI3 regulon (genes directly regulated by the seed‐specific TF ABI3). In *A. thaliana*, RNA‐Seq and ChIP‐chip data have been used to identify a set of 98 target genes that constitute the ABI3 regulon (Mönke *et al.*, 2012). We expanded our search of seed‐specific genes to include all *X. humilis* homologues of the 98 *A. thaliana* ABI3 regulon genes, several of which are not included in the Le *et al. *(2010) dataset. We identified 70 potential homologues in *X. humilis* to 62 of these genes, of which 64 (91%) were DE in one or both tissues (16 in the seed, five in the leaf and 43 in both tissues). The majority were upregulated during late seed maturation and at RWC below 60% in the leaf, and generally had very high expression levels (Figure 4b), supporting the idea that the ABI3 regulon is activated during both processes.

### LAFL regulators and ABI5 are active during seed maturation but not induced during vegetative desiccation tolerance

**Figure 5:**
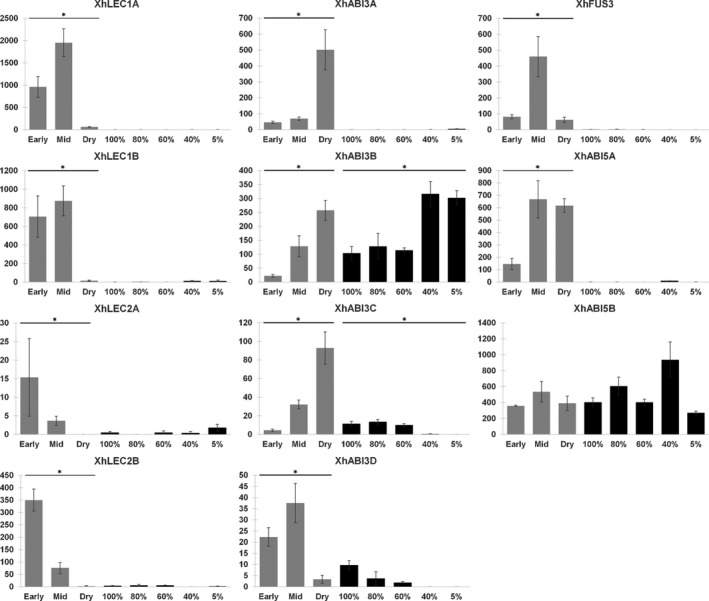
Expression of *Xerophyta humilis* LAFL and *ABI5* genes. DESeq2 normalised counts of *X. humilis* seed regulators during seed maturation (grey) and vegetative desiccation tolerance (VDT; black), marked with an asterisk if the change was significant in that tissue. Counts from both tissues are displayed on the same axis but are derived from different experiments. Error bars are standard error. (*) indicates significant at FDR < 0.01.

The induction of homologues of canonically seed‐specific genes during VDT in *X. humilis* is consistent with the hypothesis that VDT evolved through de‐repression or reactivation of seed maturation transcriptional regulators in vegetative tissue. To test this hypothesis, we queried our transcriptome for orthologues of members of the canonical LAFL regulatory network; specifically, the genes *LEC1*, *ABI3*, *FUS3* and *LEC2*, and the downstream group‐A bZIP *ABI5*. We identified one or more co‐orthologues to each of these, and the evolutionary relationships between these sequences in *X. humilis* and other plant species are shown in Figure S3. Two *X. humilis LEC1* co‐orthologues were expressed during early seed maturation and upregulated during mid‐maturation, consistent with the known role of this TF as a regulator of seed development. In contrast, these genes were barely expressed in leaf tissue irrespective of RWC, with mean normalised read counts < 10 across all RWC (Figure 5). Two putative orthologues of the monocot *LEC2*‐like genes and a putative orthologue of monocot *FUS3* were also identified in *X. humilis*. Both *XhLEC2*‐like genes were downregulated during seed maturation, while *XhFUS3* was upregulated specifically during mid‐maturation. As with the *XhLEC1* genes, the normalised read counts of *XhFUS3* and *XhLEC2* transcripts in leaves were extremely low (mean count < 6) across all RWCs (Figure 5).

Interestingly, we found that *X. humilis* possesses at least four genes with homology to *ABI3* (Figure S3), three of which (*XhABI3A‐C*) were upregulated during late seed maturation, while one (*XhABI3D*) was upregulated during earlier stages of seed development (Figure 5). *XhABI3B* was also expressed in leaves and upregulated during desiccation, but levels of the other *ABI3* transcripts were barely detectable in leaves (Figure 5). Lastly, two transcripts with similarity to the *A. thaliana* and rice *ABI5* genes were identified in *X. humilis* (Figure S3). *XhABI5A* was significantly upregulated during mid and late seed maturation but expressed at very low levels in the leaf desiccation series, while *XhABI5B* was not DE in either tissue but was consistently expressed throughout both seed maturation and VDT (Figure 5). The seed‐associated patterns of gene expression observed for the canonical LAFL TFs are consistent with a role in the regulation of seed maturation in *X. humilis* (as in other plant species) but not as regulators of the VDT response.

### Duplication of ABI3 and loss of the B3 domain in Xerophyta resurrection plants

**Figure 6:**
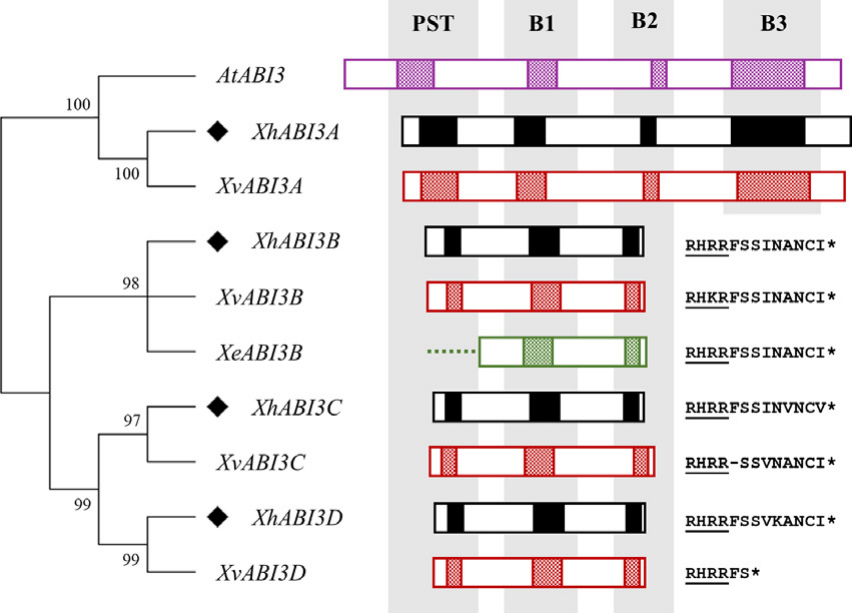
Duplication of *ABI3* in Xerophyta and loss of the B3 domain. Maximum‐likelihood phylogenetic tree showing the relationship between *AtABI3* and the predicted *ABI3* paralogues in *Xerophyta humilis, X. viscosa* and *X. elegans,* using full‐length amino acid sequences and 1000 bootstrap replicates*.* Positions of the conserved PST, B1, B2 and B3 domains are given by shaded segments. The *X. elegans ABI3B* sequence corresponds to a partial open reading frame (ORF) that is missing the N‐terminal region (dashed line). The conserved stop codon sequences for the Xerophyta‐specific *ABI3B*,* ABI3C* and *ABI3D* genes are shown on the right, with the terminal region of the B2 domain underlined. Protein alignments and phylogenetic analysis performed using Clustal Omega and RAxML.

We identified four paralogues of *ABI3* in *X. humilis*. A comparison of the protein‐coding regions of the representative transcripts for these four paralogues showed that only one, *XhABI3A*, possessed a B3 DNA‐binding domain (Figure 6). The remaining three encode a truncated ABI3 protein due to the presence of a stop codon directly after the B2 domain in their transcripts (Figure 6), including *XhABI3B* which was upregulated during both seed maturation and VDT (Figure 5). To verify that these transcripts were not artefacts from the *de novo* assembly process, 3′‐RACE was performed using primers designed to sites between the B1 and B2 domains for each *ABI3* paralogue on pooled cDNA generated from leaves and seeds. We were successful in cloning the 3′ downstream regions of the two most highly expressed *ABI3* transcripts: the B3‐containing transcript *XhABI3A* and the truncated transcript *XhABI3B*, confirming their presence and transcription termination positions (Data S2). To determine whether the increased copy number of *ABI3* is unique to *X. humilis*, we searched for similar sequences in the *X. viscosa* genome assembly using BLAST (Costa, Ligterink, *et al.*, 2017). Four *ABI3* paralogues were also detected, each on a separate scaffold of the *X. viscosa* genome, of which three also encode proteins lacking the B3 domain due to the presence of a stop codon directly after the B2 domain (Figure 6). The number and position of exons for each *X. viscosa ABI3* paralogue was determined using the complete *X. viscosa* genome assembly (Costa, Ligterink, *et al.*, 2017). The *XvABI3A* transcript has a similar structure to Arabidopsis *ABI3*, with the B1, B2 and B3 domains spread across multiple exons. In contrast, the duplicated paralogues possess only a single exon and lack the exons containing the B3 domain (Figure S4a). Expression analysis showed that of these four paralogues, only *XvABI3B* was expressed at appreciable levels in leaves and expression increased during desiccation (Figure S4b). Partial fragments to a putative *XhABI3B* orthologue in *Xerophyta elegans* were identified in a transcriptome dataset obtained from the 1000 Plants project (http://www.onekp.com/). This transcript also contained a stop codon directly after the B2 domain (Figure 6).

Xerophyta are a paleohexaploid genus, likely having undergone an ancient whole genome duplication event 55−40 Mya (Goldblatt and Poston, 1988; Alcantara *et al.*, 2018). Nonetheless, the presence of multiple *ABI3* paralogues is unusual. An analysis of B3 domain‐containing TF *ABI3* orthologues (Group 71729at3193) from OrthoDB (Kriventseva *et al.*, 2018) shows that most angiosperms and many polyploid species encode only a single copy of *ABI3*. Species with duplicated *ABI3* tended to have only two copies, though a handful of species contain more (Figure S5). *ABI3* duplication is also less prominent in monocot species compared with dicot species, being observed so far in only Xerophyta, *Sorghum bicolor*, *Musa acuminata* and the orchid species *Apostasia shenzhenica*, *Dendrobium officianale* and *Phalaenopsis equestris* (Figure S5). Only two putative ABI3s, from *A. shenzhenica* and *Nicotiana obtusifolio*, were predicted to lack a B3 domain due to a premature stop codon – though there is no additional evidence that these transcripts are expressed (Figure S5). We could not find any other similarly truncated ABI3 protein sequences in the NCBI protein database.

### Promoters of Xerophyta ABI3 regulon homologues are enriched for the ABRE but not RY motif

The majority of canonical ABI3 regulon genes were upregulated during seed maturation and VDT in *X. humilis* (Figure 4b), and also during VDT in *X. viscosa* (Costa, Ligterink, *et al.*, 2017). In stark contrast, during VDT only one LAFL paralogue – an unusual *ABI3*‐like gene lacking the B3 domain – was induced in *X. humilis* and *X. viscosa*, while the transcript encoding a full‐length ABI3 protein was barely detectable (Figures 5 and S4). The B3 domain of ABI3 recognises the RY motif (CATGCA), which is enriched in the promoters of its target genes in a range of plant species (Reidt *et al.*, 2000). To determine whether control of the ABI3 regulon in Xerophyta resurrection plants may differ from that in *A. thaliana*, we looked for evidence of altered enrichment of regulatory motifs in the promoters of ABI3 regulon genes between *A. thaliana* and *X. viscosa*, for which a complete genome is available (Costa, Ligterink, *et al.*, 2017).

**Figure 7:**
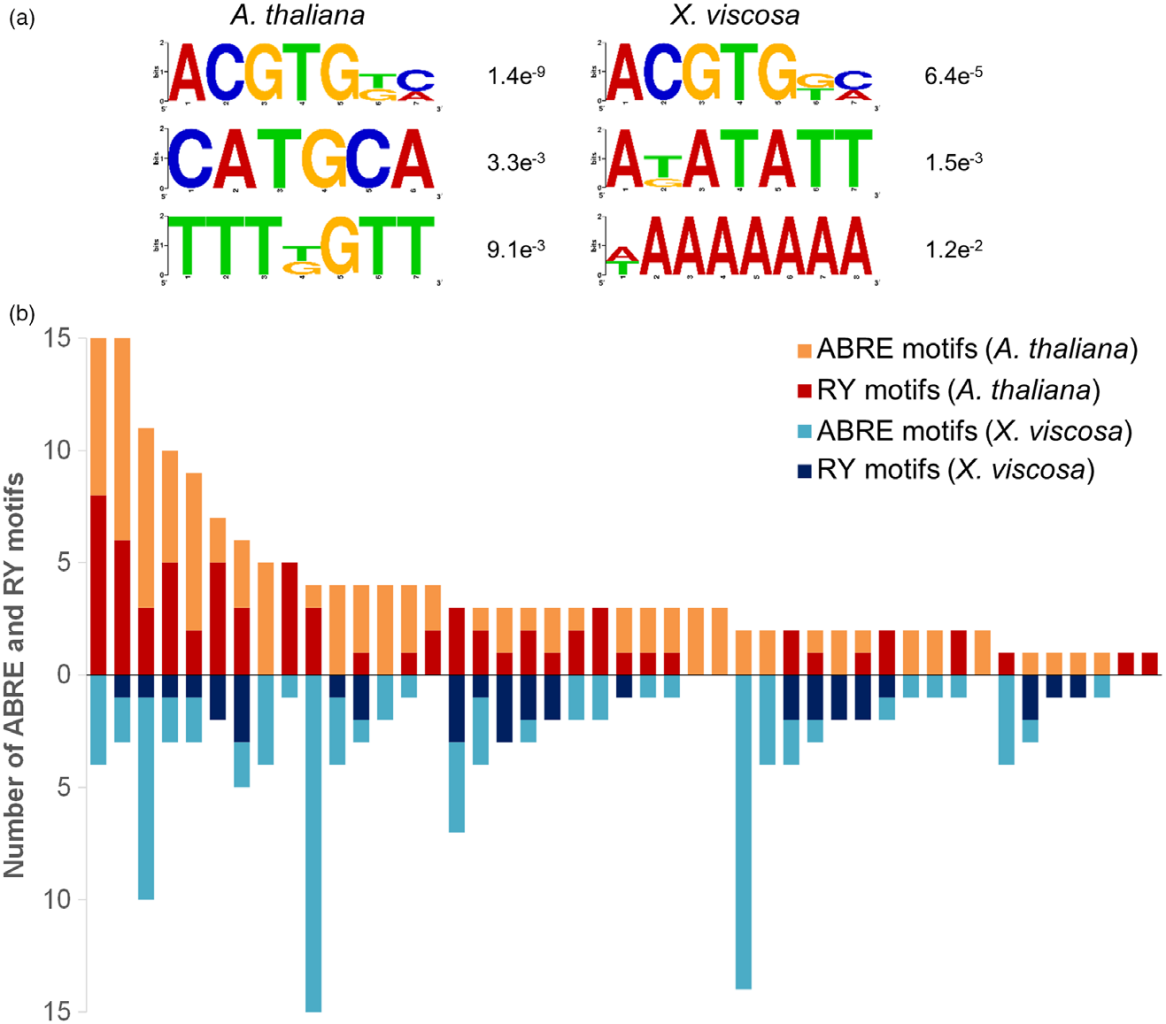
The RY and ABRE motifs in the promoters of *Arabidopisis thaliana* and *Xerophyta viscosa* ABI3 regulon genes. (a) Over‐represented motifs in the upstream 500‐bp promoter regions of the ABI3 regulon genes in both species, as determined by DREME. The ABRE (ACGTG) is enriched in both species, whereas the RY element (CATGCA) is enriched in only *A. thaliana.* (b) Total number of ABRE and RY motifs present in the 500‐bp upstream regions of *A. thaliana* ABI3 regulon genes, and all equivalent orthologues found in *X. viscosa.*

We found 105 putative homologues in *X. viscosa* to 62 of the 98 *A. thaliana* ABI3 regulon genes. Enriched motif identification was performed with DREME, using the regions 500 bp upstream of the TSS of the 62 *A. thaliana* genes and the 105 putative homologues in *X. viscosa* as input. As expected, the RY motif was enriched within the *A. thaliana* promoters, as was the ABRE (ACGTG[G/T][C/A]) (Figure 7a). A third enriched motif, TTT[G/T]GTT, shows similarity to the Pollen Box (PB) motif identified in a few pollen‐specific genes (Okada *et al.*, 2000), consistent with the fact that several ABI3 regulon genes have been shown to be expressed in stamen/maturing pollen tissue in *A. thaliana* (Mönke *et al.*, 2012). In contrast, while there was enrichment of the ABRE motif in the *X. viscosa* promoters, the RY motif was not enriched (Figure 7a). Another motif enriched in the *X. viscosa* gene promoters, similar to the heptamer sequence AGATATT, is associated with gene expression during abiotic stress (drought, heat, salt, oxidative and wounding stress) and with the expression of several stress‐related TFs, including the ABF bZIP *AtABF1* (atted.jp/data/cis/AGATATT.html; Obayashi *et al.*, 2006).

To account for the presence of duplicated co‐orthologues that may have diverged in their promoter *cis*‐element composition, we also analysed the total number of RY and ABRE motifs present within each set of genes when combined into orthogroups (defined here as a collection of *A. thaliana* and *X. viscosa* genes that have the same top blast matches to each other). The 62 *A. thaliana* genes could be divided into 45 orthogroups, and the total number of RY and ABRE motifs within the orthogroup gene promoters within 500 bp of the TSS on either strand was determined (Figure 7b). The RY motif was far more prominent in the *A. thaliana* gene promoters than those of *X. viscosa*, despite the smaller number of sequences (72 RY motifs across 62 *A. thaliana* promoters versus 37 RY motifs across 105 *X. viscosa* promoters; Figure 7b). In contrast, the ABRE featured prominently across orthogroups from both species. To test whether the 500 bp limit was too restrictive, we also investigated the distribution of RY and ABRE motifs up to 3000 bp upstream of the TSS in genes in both species. The RY element was enriched within the first 200 bp upstream of the tested *A. thaliana* genes but showed no such strong peak in *X. viscosa* (Figure S6a), while the ABRE is found within the first 200–300 bp of the promoters of both species (Figure S6b). This suggests that seed maturation genes in Xerophyta may be under the control of a different transcriptional regulatory network than in *A. thaliana*, one where the RY motif is less central.

ABI3 classically activates its target genes by binding directly to the RY element. However, there is evidence that ABI3 may also regulate gene expression through actions independent of the B3 DNA‐binding domain, for example via interaction with other TFs, including bZIPs, mediated by the B1/B2 domains (Ezcurra *et al.*, 2000; Nakamura *et al.*, 2001). Suzuki *et al.* (2014) investigated the B3‐dependent and B3‐independent activation of a small number of seed‐specific genes in transgenic *A. thaliana* by overexpression of *VP1* (the maize *ABI3* orthologue) with or without a functional B3 domain. B3‐independent genes included several oleosins, LEAs and *Responsive to Desiccation 29B*. In contrast, activation of the 2S‐albumins and *CRUCIFERIN C *(*AtCRUC*) required the presence of the B3 domain. While no 2S‐albumins were detected in the *X. humilis* transcriptome, both *XhCRUC* orthologues were only induced during seed maturation and not expressed at all in leaves (Figure S8), correlating with the lack of full‐length ABI3 (*XhABI3A*) in these tissues. In contrast, orthologues of the B3‐independent genes (two oleosins and a gene similar to *AtEM1*) were induced during late seed maturation and during VDT in leaves when only the truncated ABI3 (*XhABI3B*) is expressed (Figure S8).

### A bZIP transcription factor can drive expression from seed gene promoters in *Xerophyta humilis*

**Figure 8:**
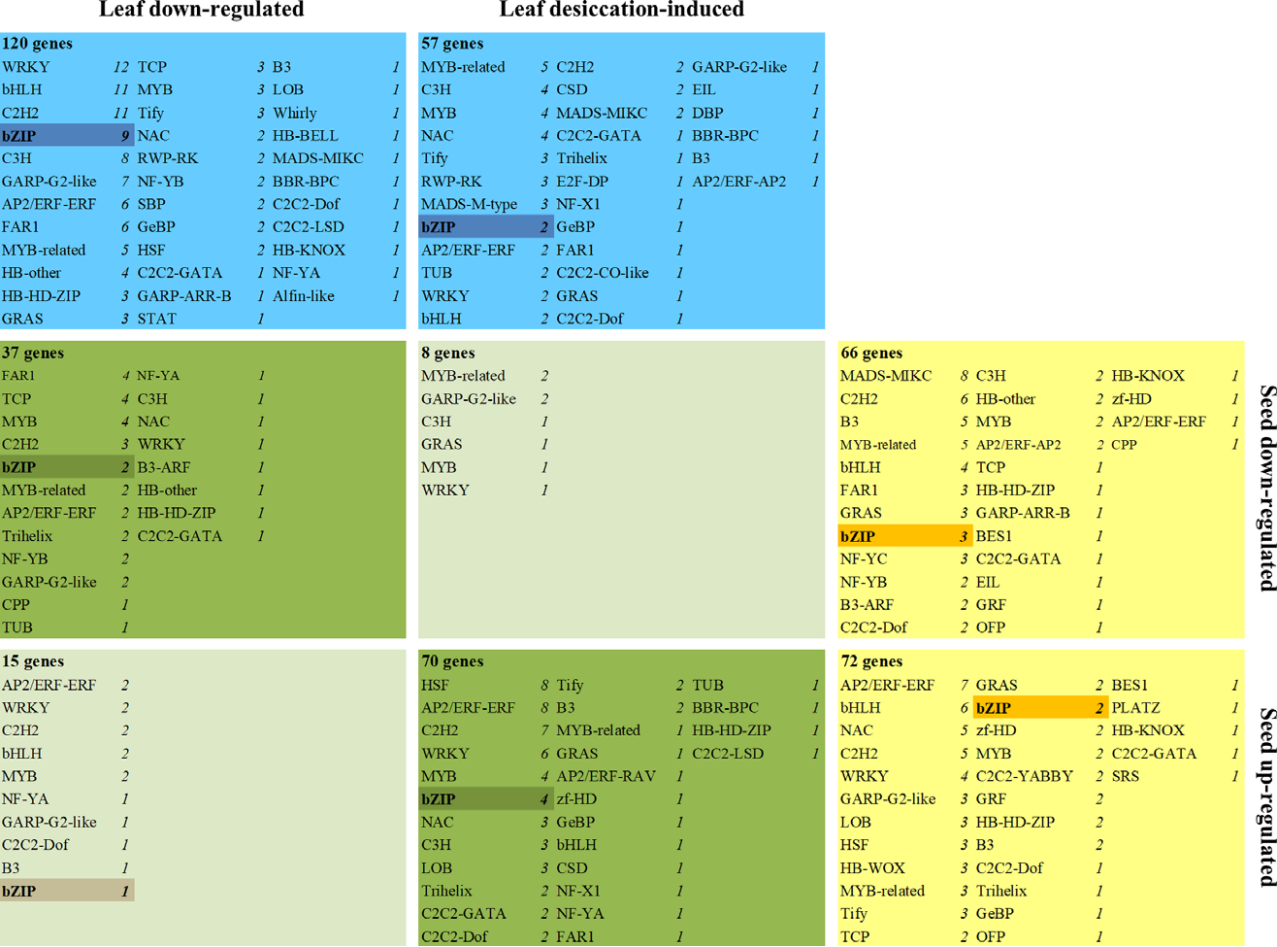
*Xerophyta humilis* transcription factors (TFs) differentially expressed (DE) during seed maturation and vegetative desiccation tolerance (VDT). Overlap between TFs expressed in seeds and leaves, using the same criteria as Figure 4, but omitting the dehydration‐induced leaf cluster that contained only a single TF. The number of genes in each group is indicated, as well as the numbers of each TF family present and the presence or absence of bZIP TFs (shaded).

As a first step in dissecting the transcriptional networks involved in seed maturation and VDT in *X. humilis*, we investigated the changes in expression of TF genes during these two processes. TFs present in the *X. humilis* transcriptome were identified using the program iTAK, resulting in the classification of 695 TF‐encoding genes across 61 families. Over 60% of these (446) were DE during seed maturation or VDT (Figure 8), with 130 DE during both processes. In general, TF families were not unique to either tissue type or biological process (Figure 8).

**Figure 9:**
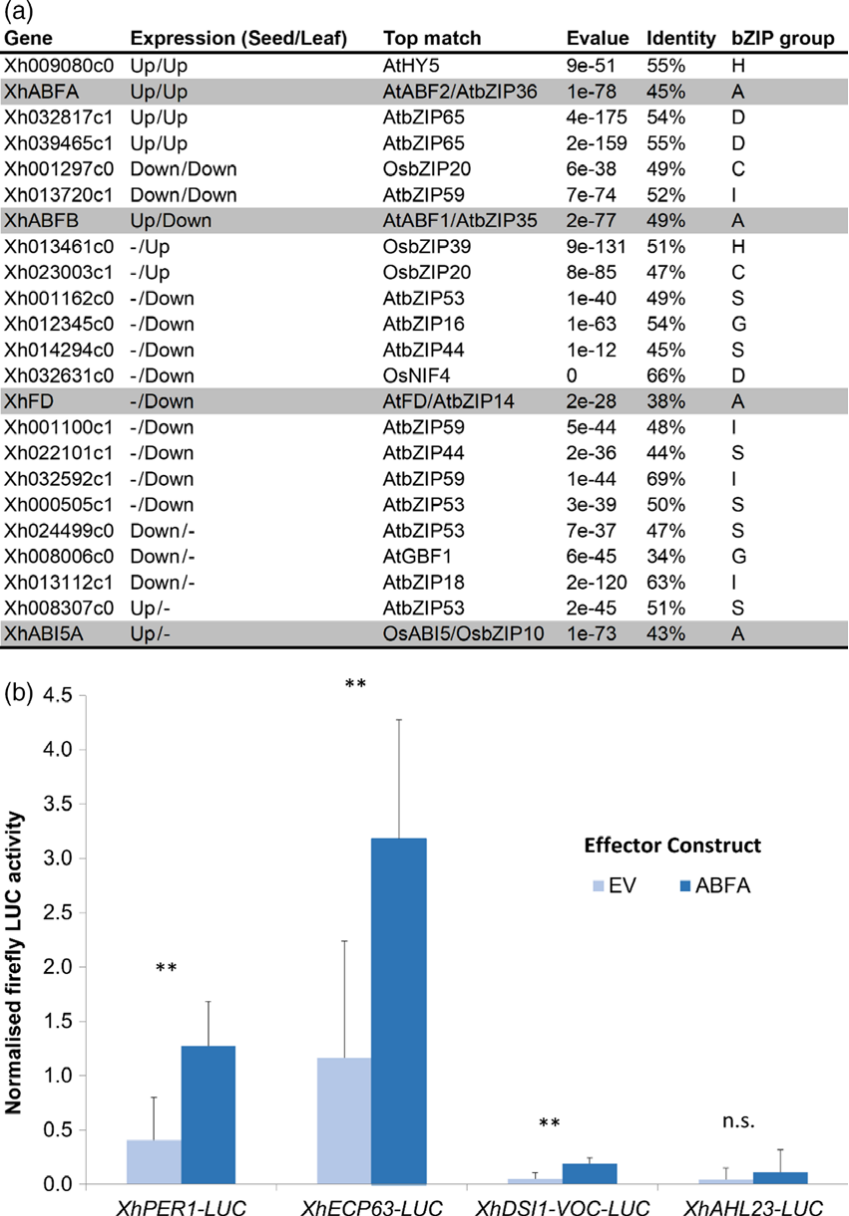
XhABFA activates transcription from ‘seed gene’ promoters. (a) Differentially regulated bZIP transcription factors (TFs) in *Xerophyta humilis* leaves and/or seeds, together with most likely bZIP group classification based on top BLASTP hit against the Swissprot database. Group A bZIPs have been shaded. (b) Protoplasts from 4‐week‐old *Arabidopsis thaliana* plants were co‐transfected with three vectors: *35S‐GFP*, *Firefly LUC* and *35S‐Renilla LUC*. The GFP vector either contained *35S‐XhABFA* or it was an empty vector ‘EV’ control. The* Firefly LUC* reporter contained one of four promoter elements: *XhPER1, XhECP63, XhDSI‐1VOC* or *XhAHL23.* Firefly LUC activity was determined 22 h post‐transfection and normalised to the geometric mean value of the Renilla LUC and GFP signals for each transfection. Values shown are mean Firefly LUC activities ± SD from nine biological repeats (except *XhECP63‐LUC* where *n* = 8 and *XhAHL23‐LUC* where *n* = 5). The *P*‐values shown are from a two‐tailed *t*‐test with Bonferroni correction (^**^ *P* < 0.01).

The ABRE was the most highly enriched motif in the promoters of *X. viscosa* seed maturation genes (Figure 7a), and ABA signalling is known to be important for the regulation of VDT (Zhang and Bartels, 2018). Gene regulation via the ABRE is controlled by bZIP TFs in *A. thaliana*, particularly those of the group A sub‐family, which includes *ABI5* (Dröge‐Laser *et al.*, 2018). Of the four group A bZIPs DE in our *X. humilis* experiments, a single TF (*XhABFA*) was highly upregulated in leaves during desiccation (< 60% RWC), and to a lesser extent in seeds after mid‐maturation (Figures 9a and S7). To determine whether XhABFA might be able to activate expression of *X. humilis* homologues of the ABI3 regulon, we used protoplast transactivation assays to test whether it was able to drive transcription from the first 350 bp of the *X. humilis PER1*,* ECP63* and *DSI‐1VOC* promoters. These genes are expressed exclusively during seed development in Arabidopsis (Yang *et al.*, 1997; Aalen, 1999; Mulako *et al.*, 2008), but are activated during seed development as well as in leaves in response to desiccation in *X. humilis* (Figure S7). *PER1* and *ECP63* are known ABI3 targets in *A. thaliana* (Haslekås *et al.*, 2003; Mönke *et al.*, 2012), and both *PER1* and *DSI‐1VOC* are expressed ectopically in homozygous *fie* (a subunit of PRC2) Arabidopsis mutants (Bouyer *et al.*, 2011). The cloned promoters of these genes have multiple ABRE elements (Figure S9). Arabidopsis protoplasts transfected with the *XhPER1‐LUC*,* XhECP63‐LUC* and *XhDSI‐1VOC‐LUC* reporter plasmids displayed significantly higher firefly luciferase (LUC) reporter activity when co‐transfected with the effector plasmid containing *XhABFA* than did those co‐transfected with the empty effector plasmid (Figure 9b). To demonstrate that transactivation by XhABFA is sequence selective, we also transfected protoplasts with a *XhAHL23*‐LUC (AT HOOK PROTEIN 23) reporter plasmid. This gene was not DE in *X. humilis* during seed maturation or VDT (Figure S7), nor does its promoter contain any ABRE elements (Figure S9). LUC activity was uniformly low in these protoplasts when co‐transfected with either the ABFA effector vector or the empty vector control, indicating that ABFA was not able to drive transcription from this promoter (Figure 9b).

## Discussion

The favoured hypothesis for the origin of VDT in angiosperms is that the gene regulatory networks that activate the DT response in orthodox seeds have been co‐opted by the vegetative stress response in resurrection plants, allowing them to mimic the protective responses used by drying embryos in their adult tissues (Costa, Cooper, *et al.*, 2017; VanBuren, 2017). Although there is ample evidence demonstrating the expression of seed maturation‐specific genes in the drying vegetative tissues of resurrection plants (Illing *et al.*, 2005; Rodriguez *et al.*, 2005; VanBuren *et al.*, 2015; Costa, Ligterink, *et al.*, 2017), it is unknown whether these genes are indeed activated as a consequence of the reactivation of conserved seed‐specific TFs. Arguments have been made for the involvement of the important seed regulators *ABI3* and *ABI5* during VDT, as these genes have been shown to be important for re‐acquisition of DT in germinating seedlings; however, neither is upregulated during VDT in resurrection plants (Dekkers *et al.*, 2015; Costa, Ligterink, *et al.*, 2015). Furthermore, no previous study has analysed the transcriptional changes during seed maturation in a resurrection plant to see if they are indeed similar to those occurring in model plants, making it impossible to determine whether the hypothesis of co‐option of seed TFs is correct.

To test this hypothesis, we assembled and quantified the dehydration transcriptomes of *X. humilis* seeds during maturation and leaves during desiccation. While there are clearly patterns of differential gene expression and biological processes that are unique to each, approximately 28% of DE genes are common to both seed maturation and VDT − with 75% of these displaying the same direction of regulation (Figure 3). Within these overlapping genes, a subset of 1637 that were upregulated during both seed maturation and late desiccation contained the majority of enriched GO terms related to abiotic stress responses, which were absent from all other gene sets (Figure 3). Our expression results also highlight a large shift in gene expression that occurs in desiccating leaves between 60% and 40% RWC, which is clearly noticeable in both the PCA plot of the RNA‐Seq data (Figure 2) and when analysing groups of VDT‐responsive genes (Figure 4). Similar observations have been made in other resurrection species, and it has been recently proposed that the physiological and gene expression changes that occur in resurrection plants around and after 40% RWC define the desiccation response (Zhang and Bartels, 2018). The clear activation of a single core set of abiotic stress response genes in both seed maturation and VDT is consistent with the argument that seed maturation and VDT share a common protection response, activated between 60% and 40% RWC in leaves and during mid/late seed maturation.

Our results provide further support for the observation that numerous genes that are seed‐specific in *A. thaliana* are upregulated during VDT in the leaves of a resurrection plant. Of the 289 seed‐specific genes identified in *A. thaliana* (Le *et al.*, 2010), we found 204 potential homologues expressed in *X. humilis*, of which 45% are DE during VDT and 50% during seed maturation, with 27% common to both processes (Figure 4a). Furthermore, the majority of homologues of the *A. thaliana* ABI3 regulon genes (59 of 70) were upregulated during late seed maturation in *X. humilis*, consistent with their role as important constituents of maturing seeds in other plants, and nearly 60% of these genes were also upregulated in leaves during VDT (Figure 4b). However, while we identified orthologues of the canonical LAFL TFs *LEC1, ABI3*, *FUS3* and *LEC2* and the bZIP *ABI5* in *X. humilis*, which were upregulated during seed maturation, the transcripts of these genes were barely detectable in leaves; instead, only transcripts for an *ABI3* paralogue lacking the B3 DNA‐binding domain (Figure 5) showed a significant increase in abundance in leaves during desiccation. These data strongly suggest that the ABI3 regulon and other seed maturation genes are activated by a different regulatory network in seeds versus leaves in *X. humilis*.

Interestingly, we found that both *X. humilis* and *X. viscosa* possess four paralogues of *ABI3* in their genomes. *Xh/XvABI3A* has all the functional domains identified in *AtABI3*, and is highly expressed during seed development, but not in leaves. However, the other three *ABI3* genes lack the B3 domain entirely due to the presence of a common stop codon, though they maintain N‐terminal acidic regions separated by a PST domain, as well as the complete B1 and B2 domains (Figure 6). We could not find similarly truncated *ABI3* genes in the NCBI protein database, but did find a partial transcript matching the truncated *XhABI3B* gene in *X. elegans* (Figure 6). The *X. elegans* RNA was sampled from young shoots, and the failure to detect either full‐length *ABI3A* or the other two truncated *ABI3* genes is consistent with the absence of these transcripts in leaves of *X. humilis*.

The *ABI3* transcript is known to be post‐transcriptionally modified in multiple species − including the formation of splice variants lacking the B3 and/or other domains (McKibbin *et al.*, 2002; Fan *et al.*, 2007; Gagete *et al.*, 2009; Sugliani *et al.*, 2010). However, our analysis of the three truncated *X. viscosa* *ABI3* transcripts indicated that they were derived from three genomic scaffolds that lack any sequences with homology to the B3 domain, indicating that these are not splicing variants but rather paralogues in which the B3 domain has been lost. Additionally, unlike full‐length *XvABI3A*, transcripts of the other three genes in *X. viscosa* contain only a single exon. Phylogenetic analysis of these genes implies that the duplication events from which they arose predates the speciation of *X. humilis* and *X. viscosa* and occurred after the mutation resulting in the truncation (Figure 6). The expression, maintenance and further duplication in Xerophyta of the *ABI3* genes bearing this mutation implies some functional activity of the encoded truncated protein and associated benefit to the ancestral lineages, possibly through neofunctionalisation of the duplicated copies. The truncated *ABI3* genes are expressed *in planta*; two of the three truncated *XhABI3* transcripts were induced during seed maturation, and one of these was also expressed in leaves and upregulated during late desiccation (Figure 5).

The B3 domain of ABI3 is known to bind to the seed‐specific RY motif, which is found in many of the promoters of seed maturation genes directly regulated by ABI3 in *A. thaliana*. A comparison of the promoter regions of the ABI3 regulon genes in both *A. thaliana* and *X. viscosa* revealed that the RY motif was diminished in the promoters of *X. viscosa* seed genes, even when accounting for gene duplications, whereas the ABRE was enriched in the promoters from both species (Figure 7). The question arises as to what role these truncated XhABI3 paralogues might play during VDT. ABI3 proteins lacking the B3 domain can still regulate gene expression, most likely via interaction with other TFs, including bZIPs, mediated by the B1/B2 domains. Maize ABI3 orthologues lacking a functional B3 domain can complement the desiccation‐intolerant phenotype of *abi3* Arabidopsis mutant seeds, and constitutive overexpression of this protein results in the upregulation of a subset of ABI3‐regulated genes (Suzuki *et al.*, 2014). Orthologues of these B3‐independent genes (two oleosins and a gene similar to *AtEM1*) were induced during VDT in *X. humilis*, when only the truncated *XhABI3B* was expressed, whereas *X. humilis CRUC* genes (orthologues of the B3‐dependent target *AtCRUC*) were induced only during seed maturation and not during VDT (Figure S8). It is thus possible that XhABI3B could be involved in regulation of gene expression during VDT despite lacking the B3 domain, although further work is required to determine if this is the case. The B1 and B2 domain of ABI3 are known to be important for interactions with ABI5 during seed maturation. However, *ABI5* is not DE during desiccation in *X. humilis* leaves. Instead, ABI3B might interact with one or more other bZIP TFs that bind to the ABRE. ABA signalling is an important element of both seed maturation and VDT (Santos‐Mendoza *et al.*, 2008; Zhang and Bartels, 2018), and *X. viscosa* ABI3 regulon promoters, while depleted for the RY motif, are still enriched for the ABRE (Figure S6). GO terms related to ABA signalling were enriched not only in the set of genes upregulated specifically during seed maturation, but also in the set upregulated during both seed maturation and vegetative desiccation (Figure 3). This is consistent with a central role for ABA signalling during these responses, and implies an important role for ABRE‐binding TFs during the acquisition of DT seeds and leaves of *X. humilis*. The *ABI5* gene and its paralogues, such as *EEL*, are known to regulate seed genes during seed maturation and, consistent with this, an *XhABI5* orthologue was upregulated at this time point (Figure 5). However, no *ABI5* orthologue was found to be upregulated during VDT. Instead a single group‐A bZIP (*XhABFA*), with similarity to the *A. thaliana* ABF TFs, was strongly induced during VDT (and to a lesser extent during seed maturation) making it a promising candidate for ABA‐dependent regulation of seed‐specific genes in the leaf tissues of *X. humilis*. Using protoplast transactivation assays we have demonstrated that XhABFA can drive expression from the *X. humilis PER1*,* ECP63* and *DSI‐1VOC* promoters (Figure 9b); the orthologues of these genes are only expressed during seed maturation in desiccation‐sensitive plants. Furthermore, experimental evidence supports *PER1* and *ECP63* as direct ABI3 target genes (Mönke *et al.*, 2012). Further experiments are required to validate the binding of XhABFA to these promoters in a chromatin‐dependent context, and to determine whether this occurs only during vegetative desiccation or also during seed maturation in *X. humilis*.

The ABF genes in *A. thaliana* constitute a semi‐redundant family of abiotic stress response factors specifically regulating the ABA response in vegetative tissues, in contrast to *ABI5*‐related genes that are expressed in embryonic tissues (Kim, 2006; Yoshida *et al.*, 2010; Yoshida *et al.*, 2015). If an *X. humilis* ABF TF is responsible for activating seed gene expression during VDT, it would suggest that at least part of the seed maturation network in Xerophyta has been captured by the vegetative stress response pathway rather than by ectopic expression of seed maturation regulators.

## Experimental Procedures 

### Plant material

*Xerophyta humilis* plants were collected from Borakalalo Nature Reserve (Northwest Province, South Africa) and transported in a desiccated state to the University of Cape Town (North West Provincial Government Permit 062 NW‐12; Cape Nature Permit AAA007‐01733). The dried plant mats were transferred to a total of three growth trays (60 × 450 × 220 mm) containing soil from the collection site and maintained in a glasshouse under ambient light conditions after rehydration.

For leaf RNA‐Seq, the plant trays were transferred to a climate‐controlled plant growth chamber (Conviron Adaptis A350) 3 weeks prior to sampling to acclimatise to the experimental conditions: 16‐h‐long day, temperature setting of 22°C, an average luminosity of 250 µmol m^−2^ sec^−1^ and watering three times a week. Undamaged leaves were sampled from clusters of plantlets across all trays at the same time mid‐morning over a period of approximately 2 weeks after the cessation of watering. Sampled leaves were split down the mid‐vein, with half used for RNA isolation and half for RWC determination. The RWC of each leaf was calculated using the following equation: RWC = (W_wet_ – W_dry_)/(W_dry_ × TW_avg_), where W_wet_ is the wet weight of the sampled leaf tissue, W_dry_ is the dry weight of the same tissue after 3 days at 60°C, and TW_avg_ is the average turgor weight of a *X. humilis* leaf. A TW_avg_ value was calculated from the average of 15 control leaves across all three trays, using the equation: TW_avg_ = (W_turgid_ – W_dry_)/W_dry_, where W_turgid_ is the turgid weight of a leaf soaked in distilled water overnight at 4°C, and W_dry_ is the dry weight of that leaf.

In order to collect *X. humilis* seed material, pollen was transferred between flowering plants with gentle brushing, and fertilised flowers were marked with cotton thread and allowed to ripen on the parent plant. Seeds were dissected from seed pods and photographed on a Nikon Stereoscope Zoom Microscope (SMZ1500). Seeds were measured using NIS‐Elements (Nikon) digital 3D imaging software. Seeds from at least three seed pods per DAF stage were imaged, with a range of 34−161 seeds measured per pod. For RNA‐Seq, seed pods were harvested at 6, 11 or 17 DAF, flash frozen in liquid nitrogen and stored at −80°C.

### RNA extraction and sequencing

RNA extraction from individual leaves was performed using the Qiagen RNeasy Plant Mini Kit following the manufacturer’s instructions. Leaf RNA extracts at each RWC determined to be of sufficient quality by gel electrophoresis and RIN analysis (BioAnalyser) were pooled to form three independent biological samples, with each pool consisting of equimolar extracts from one−five individual leaves from plants across any of the three plant trays. The leaf RWCs for the RNA‐Seq experiments were 100% (fully hydrated), 80%, 60%, 40% and 5% (fully desiccated). RNA extraction from seed tissue was performed using a modified SDS‐Trizol extraction protocol, using the entire contents of a seed pod (approximately 200 seeds, following removal of any parental tissue) as starting material (Wang *et al.*, 2012). Three biological replicates at each developmental stage (DAF6, DAF11 and DAF17) were created by combining extractions from two or three independent seed pod RNA extractions from different plants.

Purified RNA was treated with RNAstable LD (Biomatrica) and shipped to the Beijing Genomic Institute (BGI) for sequencing. Sequencing libraries were prepared using the Illumina TruSeq Stranded Total RNA with Ribo‐Zero Plant kit (Illumina), and 150 bp fragment size. Sequencing was performed over eight lanes of an Illumina flow cell using a HiSeq2000 sequencing instrument and a 90‐bp paired‐end amplification protocol (leaf RNA) or a HiSeq4000 with 150‐bp paired‐end amplification protocol (seed RNA).

### Transcriptome assembly and annotation

Assembly and assembly post‐processing were performed on a high‐performance computing cluster (Dakon) at the UCT Computational Biology Division, Cape Town, South Africa. Read quality was assessed using the FastQC software package (v0.10.1; http://www.bioinformatics.babraham.ac.uk/projects/fastqc/). For each library, Illumina adaptors and low‐quality nucleotides were removed with Trimmomatic (Bolger *et al.*, 2014), using a sliding window size of 4, a PHRED quality score cut‐off of 20 and a minimum read length of 36. Orphaned reads lacking a read pair were discarded from the dataset. Error correction of the remaining paired reads was performed using SEECER (v1.0.3; Le *et al.*, 2013) using a kmer size of 25. Although multiple assemblers were tested, *de novo* transcriptome assembly was ultimately performed using Trinity (v2.0.6; Grabherr *et al.*, 2011) in stranded mode. For transcriptome assembly, the corrected reads for both the leaf and seed libraries were combined and normalised using the Trinity *in silico* normalisation pipeline in paired‐end mode, with a kmer size of 25, a maximum coverage of 50 and a PCTS of 200.

Reads were mapped to the Trinity *de novo* transcriptome using Salmon (v0.8.1; Patro *et al.*, 2017). Contigs were initially clustered using RapClust (Srivastava *et al*., 2016), and the clustered genes were further refined using genomic information derived from a draft *X. humilis* genome (Schlebusch, 2019). The quality of the raw and clustered transcriptome assembly was calculated according to TransRate (Smith‐Unna *et al.*, 2016), while completeness was assessed using the BUSCO pipeline (Simão *et al.*, 2015).

### Transcriptome annotation

The full set of assembled transcripts was compared with known plant protein sequences with NCBI blastx (Camacho *et al.*, 2009) against local installations of the Swissprot and Uniref90 databases. The blastx parameters were tailored to suit those required for downstream Blast2GO analysis, with an e‐value cut‐off of 1e^‐6^ (https://www.blast2go.com/support/blog/22-blast2goblog/111-format-fasta-file-blast). ORF prediction was done using the Trinity suite program TransDecoder (Haas *et al.*, 2013). Representative transcripts from each gene were selected based on length of the longest predicted complete ORF or, if lacking an ORF, the longest contig sequence. Protein sequences derived from representative transcripts were used as input to a local installation of InterproScan (Zdobnov and Apweiler, 2001), and the blast results for each representative transcript as well as the identified InterPro terms were imported into Blast2GO Pro (Conesa *et al.*, 2005). Gene ontology terms for each gene were assigned from the representative transcripts using the recommended mapping/annotation settings, followed by Annex annotation augmentation.

### Differential expression and gene ontology analysis

Gene‐level expression estimation and DE analysis were performed using DESeq2 (v1.8.1; Love *et al.*, 2014). The tximport R package (Soneson *et al.*, 2015) was used to import Salmon transcript level abundance estimates and convert them to RapClust gene‐level counts (Love *et al*., 2017). DE testing was performed independently on the seed and leaf read data using a log‐likelihood ratio test with a false discovery rate cut‐off of 0.01. Only genes with an adjusted *q*‐value < 0.01 and a log_2_ fold‐change of at least 1 were considered as DE in either dataset. GO enrichment was performed using the topGO R package (Alexa and Rahnenfuhrer, 2010), using the ‘Fisher’ statistical test and ‘weight01’ algorithm. Only GO terms with an unadjusted *P*‐value of < 0.01 were considered significant.

### Quantitative polymerase chain reaction validation of RNA‐Seq data

Reverse transcriptase (RT)‐qPCR was used to validate the RNA‐Seq data for *XhABFA* and two known ABI3 regulon genes *XhPER1* and *XhCal1* (Figure S2), in leaves at five RWCs (100, 80, 60, 40 and 5%). qPCR was performed using a RotorGene RG3000A instrument (Corbett Research, Australia). Reactions consisted of 1 μL template cDNA, 5 μL Kapa SYBR FAST Universal 2 × qPCR Master Mix (Kapa Biosystems, South Africa), and 200 nm of each primer in a final volume of 10 μL. Amplification conditions included an initial step at 95°C for 3 min, followed by 40 cycles of 95°C for 5 sec, primer annealing at 60°C for 30 sec, and elongation at 72°C for 1 sec. Melt curve analysis confirmed that the individual amplified products corresponded to a single DNA fragment. The relative expression level of each gene of interest was calculated with the RotorGene 6000 series software v1.7 using the standard curve method, with normalisation to the reference gene *XhMSRB5* (peptide methionine sulfoxide reductase B5), which was not DE during leaf desiccation. Details of the primers used are given in Table S1.

### Transcriptome analysis

ABI3 regulon genes in *A. thaliana* were obtained based on the analysis performed by Mönke *et al.* (2012), and the list of seed‐specific *A. thaliana* genes as well as seed‐stage expression levels was obtained from Le *et al.* (2010). Protein, nucleotide and promoter (500 bp and 3000 bp) sequences for both lists of genes were retrieved from the TAIR online database. Homologues between these genes and proteins in *X. humilis* and *X. viscosa* were identified using blastp as described in Costa, Ligterink, *et al. *(2017) in *X. viscosa*: all hits with an e‐value within 10^‐10^ of the gene with the lowest e‐value, with an e‐value threshold cut‐off of 10^‐20^. Closely related genes that shared the same homologues were grouped into putative orthogroups for analysis (Costa *et al.*, 2017). Protein multi‐alignment was performed using Clustal Omega (Sievers and Higgins, 2014), and phylogenetic trees of specific gene families were created with RaxML (Stamatakis, 2014). *Xerophyta viscosa* promoter sequences for homologous ABI3 regulon genes were obtained from the published *X. viscosa* genome (version 3, 2017; Costa, Ligterink, *et al.*, 2017). Prediction of enriched motif sequences in *A. thaliana* and *X. viscosa* promoter sets was done using a local installation of the DREME package from the MEME Suite (Bailey, 2011), using as input the 500 bp and 3000 bp sequence upstream of the TSS of each gene.

Putative *X. humilis* TF genes were identified using a local installation of iTAK and the iTAK TF database (Zheng *et al.*, 2016). The full set of predicted best ORFs in the *X. humilis* transcriptome was used as input.

*ABI3* orthologues were identified using blast in *X. elegans* transcriptome assembly data for this species available from the 1000 Plants project (http://www.onekp.com/public_data.html; Leebens‐Mack *et al.*, 2019), and from other resurrection species using the relevant published genomes (VanBuren *et al.*, 2015, 2018; Xiao *et al.*, 2018).

### 3′‐RACE PCR

3′‐RACE was used to confirm the unusual transcript structure of the identified *XhABI3* genes. Four primers were designed, corresponding to the region between the B1 and B2 domains of each gene: (*XhABI3A*) 5′‐CGTGACCACCCAGCCGTTTT‐3′, (*XhABI3B*) 5′‐CCGTCATCCCCTGCTCCAAG‐3′, (*XhABI3C*) 5′‐TCCCTTTCCAACAACCCCGTTTC‐3′ and (*XhABI3D*) 5′‐AGCCACCGCATTTCCCAGAC‐3′. RACE PCR was run on cDNA pooled from both seed and leaf tissue, using the SMARTer 3′‐RACE Kit (Clontech) with standard controls and procedures except using only half reaction volumes. Only transcripts corresponding to *XhABI3A* and *XhABI3B* were amplified in these reactions, which were verified by Sanger sequencing.

### Analysis of *Xerophyta viscosa* RNA‐Seq data

ABI3 orthologues in the *X. viscosa* genome were identified using BLAST and the *XhABI3* sequences as queries. *XvABI3* transcript architecture was plotted using the AnnotationSketch module in GenomeTools (Gremme *et al.*, 2013). Gene expression was calculated by aligning the reads obtained from a leaf desiccation experiment (PRJNA295811; Costa, Ligterink *et al.*, 2017) to the genome using STAR and the *X. viscosa* annotation file updated to include the *XvABI3* gene coordinates. Read counts were determined using featureCounts (Liao *et al.*, 2013), and DE was analysed using DESeq2 and the same parameters as for *X. humilis*.

### Protoplast transactivation assays

The *X. humilis* RNA‐Seq assembly transcript for *XhABFA* was used to design PCR primers (Table S2) to amplify the full‐length *XhABFA* coding region from cDNA prepared from desiccating *X. humilis* leaves. The amplified PCR product was cloned into *pENTR1A* and recombined into the GATEWAY‐compatible expression vector *pUC19‐35S‐Rfa‐35S‐sGFP* (Li *et al.*, 2012) generating *pUC19‐35S‐ABFA‐35S‐sGFP.*

The promoter regions for *XhPER1*, *XhECP63*, *XhDSI‐1VOC* and *XhAHL23* were identified by aligning the transcripts for these genes, assembled from the RNA‐Seq data, to the partial genome assembly (Schlebusch, 2019; Figure S9). PCR primers (Table S2) were designed to amplify 297–347‐bp promoter regions from genomic DNA. The PCR products were cloned into the pENTR1A vector, before being recombined into the GATEWAY‐compatible Firefly luciferase vector pGWL7. All cloned promoters were sequenced in both directions to confirm their identity. PlantCare (Lescot *et al.*, 2002) was used to identify ABRE elements in these promoters, while a search for the RY element (CATGCA; Guerriero *et al.*, 2009) was done manually. Promoter images were drawn using AnnotationSketch, part of the GenomeTools package (Gremme *et al.*, 2013).

Protoplasts were isolated from 4‐week‐old *A. thaliana* grown under short‐day conditions and transfected with 10 µg of effector (pUC19‐35S‐ABFA‐35S‐sGFP), 8 µg reporter plasmid (*XhPER1‐LUC*, *XhECP63‐LUC*, *XhDSI1VOC‐LUC* or *XhAHL23‐LUC*) DNA and 2 μg of the control plasmid (pBS‐35S‐Ala‐Rluc) according to the protocol developed by the Sheen Lab (Yoo *et al.*, 2007). Firefly LUC activity 22 h post‐transfection was determined using the dual‐luciferase reporter assay (Promega) in a SpectraMax Paradigm microplate reader and normalised to the geometric mean of the GFP and Renilla luciferases signal measured in the same device. The resulting values were then scaled by dividing each value by the average of the normalised values across the whole experiment.

### Validation of promoter regions

Splinkerette PCR (Devon *et al.*, 1995) was used to independently check the integrity of the *XhPER1* and *XhDSI‐1VOC* promoter regions relative to the assembled mRNA transcripts. *Xerophyta humilis* genomic DNA was digested with EcoRI and XbaI, and ligated to Splinkerette adaptors (Devon *et al.*, 1995). Splk0 (5′‐TCGTACGAGAATCGCTGTCC‐3′) and Splk1 (5'‐ACTATCGTCGTCTCACCGCTCTC‐3') forward PCR primers were used in combination with reverse primers PER1‐gsp0 (5′‐ACCAATGATGTGCAGAGCTCGC‐3′) and PER1‐gsp1 (5'‐ACACAGCGCATTTGCTGGCC‐3') or DSI1‐gsp0 (5′‐ ACGACTCCGTCACGTCCTTCACATA G‐3′) and DSI1‐gsp1 (5′‐ ACGACTCCGTCACGTCCTTCACATA G‐3′) in nested PCR reactions, to amplify regions upstream of the coding regions for *XhPER1* and *XhDSI‐1VOC*, respectively. Splinkerette PCR products were cloned into pGEM^®^‐T Easy Vector (Promega) and sequenced by Sanger sequencing and aligned to the *X. humilis* assembly (Figure S9).

## Conflict of interests

The authors declare no conflict of interest.

## Author contributions

NI, RAI and RL conceptualised the study and designed the experiments with input from SAS and SGH. RL performed the majority of the experimental work. JP and SGH performed the protoplast transfection experiments. JP performed the 3′‐RACE and MP the RT‐qPCR validation experiments. NI characterised the seed development series, and the promoters used in the protoplast experiments. SAS worked closely with RL on bioinformatics analysis. RL curated the data and made the datasets available to the public. RL, RAI and NI wrote the manuscript with input from all authors.

### Data statement

All data referred to are included in the manuscript or supplementary materials of this manuscript.

## Supporting information


**Figure S1.** *Xerophyta humilis* transcriptome assembly scores and taxonomic annotation.

**Figure S2.** Quantitative PCR analysis of mRNA levels of *XhABFA*, *XhCAL* and *XhPER1* during desiccation of *X. humilis*.

**Figure S3.** Evolutionary relationship of LAFL genes across *X. humilis* and other species.

**Figure S4.** Conservation of ABI3 paralogues in *X. viscosa*.

**Figure S5.** Domain structure of ABI3 paralogues in angiosperms with duplicated ABI3.

**Figure S6.** Average motif distance from the TSS in *A. thaliana* and *X. viscosa* ABI3 regulon genes.

**Figure S7.** Expression of *XhABFA* and putative target genes.

**Figure S8.** Expression of several *X. humilis* seed genes.

**Figure S9.** Summary of promoter regions used in protoplast experiments.

**Table S1. **Primers for quantitative PCR validation of RNA‐Seq data.

**Table S2. **PCR primers to amplify the *XhABFA* coding region, or promoter regions for *XhPER1*, *XhECP63*, *XhDSI‐1VOC* or *XhAHL23* for cloning into vectors for plant protoplast experiments.

**Data S1.** Expression data for genes analysed in Figure 4.

**Data S2.** *ABI3* 3’‐RACE sequences.

## Data Availability

Raw sequence reads have been submitted to the SRA at NCBI BioProject ID PRJNA505754. Supplementary data including transcriptome sequences, representative transcript and protein sequences, gene functional annotations and gene expression values can be found at https://doi.org/10.6084/m9.figshare.c.4691234

## References

[tpj14596-bib-0001] Aalen, R.B. (1999) Peroxiredoxin antioxidants in seed physiology. Seed Sci. Res. 9, 285–295.

[tpj14596-bib-0002] Alcantara, S. , Ree, R.H. and Mello‐Silva, R. (2018) Accelerated diversification and functional trait evolution in Velloziaceae reveal new insights into the origins of the campos rupestres’ exceptional floristic richness. Ann. Bot. 122, 165–180. 29800276 10.1093/aob/mcy063 PMC6025242

[tpj14596-bib-0003] Alexa, A. and Rahnenfuhrer, J. (2010) topGO: enrichment analysis for gene ontology. R Packag. version, 2.

[tpj14596-bib-0004] Alpert, P. (2005) The limits and frontiers of desiccation‐tolerant life. Integr. Comp. Biol. 45, 685–695. 21676818 10.1093/icb/45.5.685

[tpj14596-bib-0005] Angelovici, R. , Galili, G. , Fernie, A.R. and Fait, A. (2010) Seed desiccation: a bridge between maturation and germination. Trends Plant Sci. 15, 211–218. 20138563 10.1016/j.tplants.2010.01.003

[tpj14596-bib-0006] Bailey, T.L. (2011) DREME: motif discovery in transcription factor ChIP‐seq data. Bioinformatics, 27, 1653–1659. 21543442 10.1093/bioinformatics/btr261 PMC3106199

[tpj14596-bib-0007] Behnke, H.D. , Hummel, E. , Hillmer, S. , Sauer‐Gürth, H. , Gonzalez, J. and Wink, M. (2013) A revision of African Velloziaceae based on leaf anatomy characters and *rbcL* nucleotide sequences. Bot. J. Linn. Soc. 172, 22–94.

[tpj14596-bib-0008] Bewley, J.D. and Black, M.J. (1994) Seeds: Physiology of Development and Germination. New York, NY: Plenum Press.

[tpj14596-bib-0009] Bolger, A.M. , Lohse, M. and Usadel, B. (2014) Trimmomatic: a flexible trimmer for Illumina sequence data. Bioinformatics, 30, 2114–2120. 24695404 10.1093/bioinformatics/btu170 PMC4103590

[tpj14596-bib-0010] Bouyer, D. , Roudier, F. , Heese, M. ***et al*** (2011) Polycomb repressive complex 2 controls the embryo‐to‐seedling phase transition. PLoS Genet. 7, e1002014. 21423668 10.1371/journal.pgen.1002014 PMC3053347

[tpj14596-bib-0011] Bruggink, T. and van der Toorn, P. (1995) Induction of desiccation tolerance in germinated seeds. Seed Sci. Res. 5, 1–4.

[tpj14596-bib-0012] Bruggink, T. and van der Toorn, P. (1997) Induction of desiccation tolerance in germinated impatiens seeds enables their practical use In Basic and Applied Aspects of Seed Biology (Ellis R.H., Black M., Murdoch A.J. and Hong T.D., eds.). Dordrecht: Springer, 30, pp. 461–467.

[tpj14596-bib-0013] Buitink, J. , Leger, J.J. , Guisle, I. ***et al*** (2006) Transcriptome profiling uncovers metabolic and regulatory processes occurring during the transition from desiccation‐sensitive to desiccation‐tolerant stages in *Medicago truncatula* seeds. Plant J. 47, 735–750. 16923015 10.1111/j.1365-313X.2006.02822.x

[tpj14596-bib-0017] Camacho, C. , Coulouris, G. , Avagyan, V. , Ma, N. , Papadopoulos, J. , Bealer, K. and Madden, T.L. (2009) BLAST+: architecture and applications. BMC Bioinformatics, 10, 421. 20003500 10.1186/1471-2105-10-421 PMC2803857

[tpj14596-bib-0018] Carbonero, P. , Iglesias‐Fernandez, R. and Vicente‐Carbajosa, J. (2016) The AFL subfamily of B3 transcription factors: evolution and function in angiosperm seeds. J. Exp. Bot. 68, 871–880. 10.1093/jxb/erw458 28007955

[tpj14596-bib-0019] Conesa, A. , Götz, S. , García‐Gómez, J.M. , Terol, J. , Talón, M. and Robles, M. (2005) Blast2GO: a universal tool for annotation, visualization and analysis in functional genomics research. Bioinformatics, 21, 3674–3676. 16081474 10.1093/bioinformatics/bti610

[tpj14596-bib-0020] Costa, M.‐C.D. , Cooper, K. , Hilhorst, H.W.M. and Farrant, J.M. (2017) Orthodox seeds and resurrection plants: two of a kind? Plant Physiol. 175, 00760.2017. 10.1104/pp.17.00760 PMC5619911 28851758

[tpj14596-bib-0021] Costa, M.‐C.D. , Ligterink, W. , Derks, M.F.L. ***et al*** (2017) A footprint of desiccation tolerance in the genome of *Xerophyta viscosa* . Nat. Plants, 3, 17 038. 10.1038/nplants.2017.38 28346448

[tpj14596-bib-0022] Dam, S. , Laursen, B.S. , Ørnfelt, J.H. ***et al*** (2009) The proteome of seed development in the model legume *Lotus japonicus* . Plant Physiol. 149, 1325–1340. 19129418 10.1104/pp.108.133405 PMC2649391

[tpj14596-bib-0023] Dekkers, B.J.W. , Costa, M.C.D. , Maia, J. , Bentsink, L. , Ligterink, W. and Hilhorst, H.W.M. (2015) Acquisition and loss of desiccation tolerance in seeds: from experimental model to biological relevance. Planta, 241, 563–77. 25567203 10.1007/s00425-014-2240-x

[tpj14596-bib-0024] Devon, R.S. , Porteous, D.J. and Brookes, A.J. (1995) Splinkerettes–improved vectorettes for greater efficiency in PCR walking. Nucleic Acids Res. 23, 1644. 7784225 10.1093/nar/23.9.1644 PMC306912

[tpj14596-bib-0025] Dinakar, C. and Bartels, D. (2013) Desiccation tolerance in resurrection plants: new insights from transcriptome, proteome and metabolome analysis. Front. Plant Sci. 4, 482. 24348488 10.3389/fpls.2013.00482 PMC3842845

[tpj14596-bib-0026] Dröge‐Laser, W. , Snoek, B.L. , Snel, B. and Weiste, C. (2018) The Arabidopsis bZIP transcription factor family—an update. Curr. Opin. Plant Biol. 45, 36–49. 29860175 10.1016/j.pbi.2018.05.001

[tpj14596-bib-0027] Ezcurra, I. , Wycliffe, P. , Nehlin, L. , Ellerstrom, M. and Rask, L. (2000) Transactivation of the *Brassica napus* napin promoter by ABI3 requires interaction of the conserved B2 and B3 domains of ABI3 with different *cis*‐elements: B2 mediates activation through an ABRE, whereas B3 interacts with an RY/G‐box. Plant J. 24, 57–66. 11029704 10.1046/j.1365-313x.2000.00857.x

[tpj14596-bib-0028] Fan, J. , Niu, X. , Wang, Y. , Ren, G. , Zhuo, T. , Yang, Y. , Lu, B.‐R. and Liu, Y. (2007) Short, direct repeats (SDRs)‐mediated post‐transcriptional processing of a transcription factor gene *OsVP1* in rice (*Oryza sativa*). J. Exp. Bot. 58, 3811–3817. 18057047 10.1093/jxb/erm231

[tpj14596-bib-0029] Farrant, J.M. , Bailly, C. , Leymarie, J. , Hamman, B. , Côme, D. and Corbineau, F. (2004) Wheat seedlings as a model to understand desiccation tolerance and sensitivity. Physiol. Plant. 120, 563–574. 15032818 10.1111/j.0031-9317.2004.0281.x

[tpj14596-bib-0030] Footitt, S. , Ingouff, M. , Clapham, D. and von Arnold, S. (2003) Expression of the viviparous 1 (Pavp1) and p34cdc2 protein kinase (cdc2Pa) genes during somatic embryogenesis in Norway spruce (*Picea abies* [L.] Karst). J. Exp. Bot. 54, 1711–1719. 12754264 10.1093/jxb/erg178

[tpj14596-bib-0031] Gaff, D.F. and Oliver, M. (2013) The evolution of desiccation tolerance in angiosperm plants: a rare yet common phenomenon. Funct. Plant Biol. 40, 315–328. 10.1071/FP12321 32481110

[tpj14596-bib-0032] Gagete, A.P. , Riera, M. , Franco, L. and Rodrigo, M.I. (2009) Functional analysis of the isoforms of an ABI3‐like factor of *Pisum sativum* generated by alternative splicing. J. Exp. Bot. 60, 1703–14. 19261920 10.1093/jxb/erp038 PMC2671620

[tpj14596-bib-0033] Garg, R. , Singh, V.K. , Rajkumar, M.S. , Kumar, V. and Jain, M. (2017) Global transcriptome and coexpression network analyses reveal cultivar‐specific molecular signatures associated with seed development and seed size/weight determination in chickpea. Plant J. 91, 1088–1107. 28640939 10.1111/tpj.13621

[tpj14596-bib-0034] Gechev, T.S. , Benina, M. , Obata, T. ***et al*** (2013) Molecular mechanisms of desiccation tolerance in the resurrection glacial relic *Haberlea rhodopensis* . Cell. Mol. Life Sci. 70, 689–709. 22996258 10.1007/s00018-012-1155-6 PMC11113823

[tpj14596-bib-0035] Giraudat, J. , Hauge, B.M. , Valon, C. , Smalle, J. , Parcy, F. and Goodman, H.M. (1992) Isolation of the Arabidopsis *ABI3* gene by positional cloning. Plant Cell, 4, 1251–1261. 1359917 10.1105/tpc.4.10.1251 PMC160212

[tpj14596-bib-0036] Goldblatt, P. and Poston, M.E. (1988) Observations on the chromosome cytology of Velloziaceae. Ann. Missouri Bot. Gard. 192–195.

[tpj14596-bib-0037] Grabherr, M.G. , Haas, B.J. , Yassour, M. ***et al*** (2011) Full‐length transcriptome assembly from RNA‐Seq data without a reference genome. Nat. Biotechnol. 29, 644–652. 21572440 10.1038/nbt.1883 PMC3571712

[tpj14596-bib-0038] Gremme, G. , Steinbiss, S. and Kurtz, S. (2013) GenomeTools: a comprehensive software library for efficient processing of structured genome annotations. IEEE/ACM Trans. Comput. Biol. Bioinforma. 10, 645–656. 10.1109/TCBB.2013.68 24091398

[tpj14596-bib-0039] Guerriero, G. , Martin, N. , Golovko, A. , Sundström, J.F. , Rask, L. and Ezcurra, I. (2009) The RY/Sph element mediates transcriptional repression of maturation genes from late maturation to early seedling growth. New Phytol. 184, 552–565. 19659659 10.1111/j.1469-8137.2009.02977.x

[tpj14596-bib-0040] Haas, B.J. , Papanicolaou, A. , Yassour, M. ***et al.*** (2013) De novo transcript sequence reconstruction from RNA-seq using the Trinity platform for reference generation and analysis. Nat. Protoc. 8, 1494–1512. 23845962 10.1038/nprot.2013.084 PMC3875132

[tpj14596-bib-0041] Haslekås, C. , Grini, P.E. , Nordgard, S.H. , Thorstensen, T. , Viken, M.K. , Nygaard, V. and Aalen, R.B. (2003) ABI3 mediates expression of the peroxiredoxin antioxidant *AtPER1* gene and induction by oxidative stress. Plant Mol. Biol. 53, 313–326. 14750521 10.1023/b:plan.0000006937.21343.2a

[tpj14596-bib-0042] Hattori, T. , Terada, T. and Hamasuna, S.T. (1994) Sequence and functional analyses of the rice gene homologous to the maize Vp1. Plant Mol. Biol. 24, 805–810. 8193305 10.1007/BF00029862

[tpj14596-bib-0043] Illing, N. , Denby, K.J. , Collett, H. , Shen, A. and Farrant, J.M. (2005) The signature of seeds in resurrection plants: a molecular and physiological comparison of desiccation tolerance in seeds and vegetative tissues. Integr. Comp. Biol. 45, 771–787. 21676829 10.1093/icb/45.5.771

[tpj14596-bib-0044] Ingle, R.A. , Collett, H. , Cooper, K. , Takahashi, Y. , Farrant, J.M. and Illing, N. (2008) Chloroplast biogenesis during rehydration of the resurrection plant *Xerophyta humilis*: parallels to the etioplast‐chloroplast transition. Plant. Cell Environ. 31, 1813–1824. 18771571 10.1111/j.1365-3040.2008.01887.x

[tpj14596-bib-0045] Khandelwal, A. , Cho, S.H. , Marella, H. , Sakata, Y. , Perroud, P.‐F. , Pan, A. and Quatrano, R.S. (2010) Role of ABA and ABI3 in desiccation tolerance. Science, 327, 546. 20110497 10.1126/science.1183672

[tpj14596-bib-0046] Kim, S.Y. (2006) The role of ABF family bZIP class transcription factors in stress response. Physiol. Plant. 126, 519–527.

[tpj14596-bib-0047] Komatsu, K. , Suzuki, N. , Kuwamura, M. ***et al*** (2013) Group A PP2Cs evolved in land plants as key regulators of intrinsic desiccation tolerance. Nat. Commun. 4, 2219. 23900426 10.1038/ncomms3219 PMC3731658

[tpj14596-bib-0048] Kriventseva, E.V. , Kuznetsov, D. , Tegenfeldt, F. , Manni, M. , Dias, R. , Simão, F.A. and Zdobnov, E.M. (2018) OrthoDB v10: sampling the diversity of animal, plant, fungal, protist, bacterial and viral genomes for evolutionary and functional annotations of orthologs. Nucleic Acids Res. 47, D807–D811. 10.1093/nar/gky1053 PMC6323947 30395283

[tpj14596-bib-0049] Le, B.H. , Cheng, C. , Bui, A.Q. ***et al*** (2010) Global analysis of gene activity during Arabidopsis seed development and identification of seed‐specific transcription factors. Proc. Natl Acad. Sci. USA, 107, 8063–8070. 20385809 10.1073/pnas.1003530107 PMC2889569

[tpj14596-bib-0050] Le, H.‐S. , Schulz, M.H. , McCauley, B.M. , Hinman, V.F. and Bar‐Joseph, Z. (2013) Probabilistic error correction for RNA sequencing. Nucleic Acids Res. 41, e109. 23558750 10.1093/nar/gkt215 PMC3664804

[tpj14596-bib-0051] Leebens-Mack, J.H. , Barker, M.S. , Carpenter, E.J. ***et al*** (2019) One thousand plant transcriptomes and the phylogenomics of green plants. Nature, 574, 679–685. 31645766 10.1038/s41586-019-1693-2 PMC6872490

[tpj14596-bib-0052] Leprince, O. , Pellizzaro, A. , Berriri, S. and Buitink, J. (2017) Late seed maturation: drying without dying. J. Exp. Bot. 68, 827–841. 28391329 10.1093/jxb/erw363

[tpj14596-bib-0053] Lescot, M. , Déhais, P. , Thijs, G. , Marchal, K. , Moreau, Y. , de Peer, Y. , Rouzé, P. and Rombauts, S. (2002) PlantCARE, a database of plant cis‐acting regulatory elements and a portal to tools for in silico analysis of promoter sequences. Nucleic Acids Res. 30, 325–327. 11752327 10.1093/nar/30.1.325 PMC99092

[tpj14596-bib-0054] Li, Q. , Lin, Y.‐C. , Sun, Y.‐H. , Song, J. , Chen, H. , Zhang, X.‐H. , Sederoff, R.R. and Chiang, V.L. (2012) Splice variant of the SND1 transcription factor is a dominant negative of *SND1* members and their regulation in *Populus trichocarpa* . Proc. Natl Acad. Sci. USA, 109, 14 699–14 704. 10.1073/pnas.1212977109 PMC3437841 22915581

[tpj14596-bib-0055] Liao, Y. , Smyth, G.K. and Shi, W. (2013) featureCounts: an efficient general purpose program for assigning sequence reads to genomic features. Bioinformatics, 30, 923–930. 24227677 10.1093/bioinformatics/btt656

[tpj14596-bib-0056] Lopez‐Molina, L. , Mongrand, S. and Chua, N.H. (2001) A postgermination developmental arrest checkpoint is mediated by abscisic acid and requires the ABI5 transcription factor in Arabidopsis. Proc. Natl Acad. Sci. USA, 98, 4782–4787. 11287670 10.1073/pnas.081594298 PMC31911

[tpj14596-bib-0057] Love, M.I. , Huber, W. and Anders, S. (2014) Moderated estimation of fold change and dispersion for RNA‐seq data with DESeq2. Genome Biol. 15, 550. 25516281 10.1186/s13059-014-0550-8 PMC4302049

[tpj14596-bib-0058] Love, M.I. , Anders, S. and Huber, W. (2017) Analyzing RNA‐seq data with DESeq2. *R Packag. Ref. Man*.

[tpj14596-bib-0059] Lyall, R. , Ingle, R.A. and Illing, N. (2014) The window of desiccation tolerance shown by early‐stage germinating seedlings remains open in the resurrection plant, Xerophyta viscosa. PLoS One, 9, e93093. 24667896 10.1371/journal.pone.0093093 PMC3965527

[tpj14596-bib-0060] Maia, J. (2014) Unravelling desiccation tolerance in germinated Arabidopsis seeds. Wageningen University https://edepot.wur.nl/302759

[tpj14596-bib-0061] Maia, J. , Dekkers, B.J.W. , Provart, N.J. , Ligterink, W. and Hihorst, H.W.M. (2011) The re‐establishment of desiccation tolerance in germinated *Arabidopsis thaliana* seeds and its associated transcriptome. PLoS ONE, 6, 1–11. 10.1371/journal.pone.0029123 PMC3237594 22195004

[tpj14596-bib-0062] Marella, H.H. , Sakata, Y. and Quatrano, R.S. (2006) Characterization and functional analysis of *ABSCISIC ACID INSENSITIVE3‐like* genes from *Physcomitrella patens* . Plant J. 46, 1032–1044. 16805735 10.1111/j.1365-313X.2006.02764.x

[tpj14596-bib-0063] McCarty, D.R. , Hattori, T. , Carson, C.B. , Vasil, V. , Lazar, M. and Vasil, I.K. (1991) The *Viviparous‐1* developmental gene of maize encodes a novel transcriptional activator. Cell, 66, 895–905. 1889090 10.1016/0092-8674(91)90436-3

[tpj14596-bib-0064] McKibbin, R.S. , Wilkinson, M.D. , Bailey, P.C. , Flintham, J.E. , Andrew, L.M. , Lazzeri, P.A. , Gale, M.D. , Lenton, J.R. and Holdsworth, M.J. (2002) Transcripts of *Vp‐1* homeologues are misspliced in modern wheat and ancestral species. Proc. Natl. Acad. Sci. USA, 99, 10 203–10 208. 12119408 10.1073/pnas.152318599 PMC126648

[tpj14596-bib-0065] Mönke, G. , Seifert, M. , Keilwagen, J. ***et al*** (2012) Toward the identification and regulation of the *Arabidopsis thaliana* ABI3 regulon. Nucleic Acids Res. 40, 8240–8254. 22730287 10.1093/nar/gks594 PMC3458547

[tpj14596-bib-0066] Mulako, I. , Farrant, J.M. , Collett, H. and Illing, N. (2008) Expression of Xhdsi‐1VOC, a novel member of the vicinal oxygen chelate (VOC) metalloenzyme superfamily, is up‐regulated in leaves and roots during desiccation in the resurrection plant *Xerophyta humilis* (Bak) Dur and Schinz. J. Exp. Bot. 59, 3885–3901. 18791196 10.1093/jxb/ern226 PMC2576639

[tpj14596-bib-0067] Nakamura, S. , Lynch, T.J. and Finkelstein, R.R. (2001) Physical interactions between ABA response loci of Arabidopsis. Plant J. 26, 627–635. 11489176 10.1046/j.1365-313x.2001.01069.x

[tpj14596-bib-0068] Nambara, E. , McCourt, P. and Naito, S. (1995) A regulatory role for the *ABI3* gene in the establishment of embryo maturation in *Arabidopsis thaliana* . Development, 121, 629–636.

[tpj14596-bib-0069] North, H. , Baud, S. , Debeaujon, I. ***et al*** (2010) Arabidopsis seed secrets unravelled after a decade of genetic and omics‐driven research. Plant J. 61, 971–981. 20409271 10.1111/j.1365-313X.2009.04095.x

[tpj14596-bib-0070] Obayashi, T. , Kinoshita, K. , Nakai, K. , Shibaoka, M. , Hayashi, S. , Saeki, M. , Shibata, D. , Saito, K. and Ohta, H. (2006) ATTED‐II: a database of co‐expressed genes and cis elements for identifying co‐regulated gene groups in Arabidopsis. Nucleic Acids Res. 35, D863–D869. 17130150 10.1093/nar/gkl783 PMC1716726

[tpj14596-bib-0071] Okada, T. , Sasaki, Y. , Ohta, R. , Onozuka, N. and Toriyama, K. (2000) Expression of *Bra r 1* gene in transgenic tobacco and *Bra r 1* promoter activity in pollen of various plant species. Plant Cell Physiol. 41, 757–766. 10945346 10.1093/pcp/41.6.757

[tpj14596-bib-0072] Oliver, M. , Tuba, Z. and Mishler, B. (2000) The evolution of vegetative desiccation tolerance in land plants. Plant Ecol. 85–100.

[tpj14596-bib-0073] Oliver, M.J. , Velten, J. and Mishler, B.D. (2005) Desiccation tolerance in bryophytes: a reflection of the primitive strategy for plant survival in dehydrating habitats? Integr. Comp. Biol. 45, 788–799. 21676830 10.1093/icb/45.5.788

[tpj14596-bib-0074] Patro, R. , Duggal, G. , Love, M.I. , Irizarry, R.A. and Kingsford, C. (2017) Salmon provides fast and bias‐aware quantification of transcript expression. Nat. Methods, 14, 417. 28263959 10.1038/nmeth.4197 PMC5600148

[tpj14596-bib-0075] Rakić, T. , Lazarević, M. , Jovanović, Z.S. , Radović, S. , Siljak‐Yakovlev, S. , Stevanović, B. and Stevanović, V. (2014) Resurrection plants of the genus Ramonda: prospective survival strategies ‐ unlock further capacity of adaptation, or embark on the path of evolution? Front. Plant Sci. 4, 550. 24454318 10.3389/fpls.2013.00550 PMC3887321

[tpj14596-bib-0076] Reidt, W. , Wohlfarth, T. , Ellerstrom, M. , Czihal, A. , Tewes, A. , Ezcurra, I. , Rask, L. and Baumlein, H. (2000) Gene regulation during late embryogenesis: the RY motif of maturation‐specific gene promoters is a direct target of the FUS3 gene product. Plant J. 21, 401–408. 10758492 10.1046/j.1365-313x.2000.00686.x

[tpj14596-bib-0077] Rodriguez, M.C.S. , Edsgärd, D. , Hussain, S.S. , Alquezar, D. , Rasmussen, M. , Gilbert, T. , Nielsen, B.H. , Bartels, D. and Mundy, J. (2010) Transcriptomes of the desiccation‐tolerant resurrection plant *Craterostigma plantagineum* . Plant J. 63, 212–228. 20444235 10.1111/j.1365-313X.2010.04243.x

[tpj14596-bib-0078] Santos‐Mendoza, M. , Dubreucq, B. , Baud, S. , Parcy, F. , Caboche, M. and Lepiniec, L. (2008) Deciphering gene regulatory networks that control seed development and maturation in Arabidopsis. Plant J. 54, 608–620. 18476867 10.1111/j.1365-313X.2008.03461.x

[tpj14596-bib-0079] Sasnauskas, G. , Manakova, E. , Lapenas, K. , Kauneckaite, K. and Siksnys, V. (2018) DNA recognition by Arabidopsis transcription factors ABI 3 and NGA 1. FEBS J. 285, 4041–4059. 30183137 10.1111/febs.14649

[tpj14596-bib-0080] Schlebusch, S.A. (2019) Events that shape genomes. University of Cape Town Available at: https://open.uct.ac.za/handle/11427/29115.

[tpj14596-bib-0081] Shiota, H. , Satoh, R. , Watabe, K. , Harada, H. and Kamada, H. (1998) C‐ABI3, the carrot homologue of the Arabidopsis ABI3, is expressed during both zygotic and somatic embryogenesis and functions in the regulation of embryo‐specific ABA‐inducible genes. Plant Cell Physiol. 39, 1184–1193. 9891417 10.1093/oxfordjournals.pcp.a029319

[tpj14596-bib-0082] Sievers, F. and Higgins, D.G. (2014) Clustal Omega, accurate alignment of very large numbers of sequences In Multiple Sequence Alignment Methods. Methods in Molecular Biology (Methods and Protocols) (Russell D., ed.). Totowa, NJ: Humana Press, 1079, pp. 105–116. 10.1007/978-1-62703-646-7_6 24170397

[tpj14596-bib-0083] Simão, F.A. , Waterhouse, R.M. , Ioannidis, P. , Kriventseva, E.V. and Zdobnov, E.M. (2015) BUSCO: assessing genome assembly and annotation completeness with single‐copy orthologs. Bioinformatics, 31, 3210–3212. 26059717 10.1093/bioinformatics/btv351

[tpj14596-bib-0084] Smith‐Unna, R. , Boursnell, C. , Patro, R. , Hibberd, J.M. and Kelly, S. (2016) TransRate: reference‐free quality assessment of de novo transcriptome assemblies. Genome Res. 26, 1134–1144. 27252236 10.1101/gr.196469.115 PMC4971766

[tpj14596-bib-0085] Soneson, C. , Love, M.I. and Robinson, M.D. (2015) Differential analyses for RNA‐seq: transcript‐level estimates improve gene‐level inferences. F1000Research, 4, 1521. 26925227 10.12688/f1000research.7563.1 PMC4712774

[tpj14596-bib-0086] Srivastava, A. , Sarkar, H. , Malik, L. and Patro, R. (2016) Accurate, fast and lightweight clustering of de novo transcriptomes using fragment equivalence classes. *arXiv Prepr. arXiv1604.03250*.

[tpj14596-bib-0087] Stamatakis, A. (2014) RAxML version 8: a tool for phylogenetic analysis and post‐analysis of large phylogenies. Bioinformatics, 30, 1312–1313. 24451623 10.1093/bioinformatics/btu033 PMC3998144

[tpj14596-bib-0088] Sugliani, M. , Brambilla, V. , Clerkx, E.J.M. , Koornneef, M. and Soppe, W.J.J. (2010) The conserved splicing factor SUA controls alternative splicing of the developmental regulator *ABI3* in Arabidopsis. Plant Cell, 221, 1936–1946. 10.1105/tpc.110.074674 PMC2910958 20525852

[tpj14596-bib-0089] Suzuki, M. , Kao, C.Y. and McCarty, D.R. (1997) The conserved B3 domain of VIVIPAROUS1 has a cooperative DNA binding activity. Plant Cell, 9, 799–807. 9165754 10.1105/tpc.9.5.799 PMC156957

[tpj14596-bib-0090] Suzuki, M. , Kao, C.‐Y. , Cocciolone, S. and McCarty, D.R. (2001) Maize VP1 complements Arabidopsis *abi3* and confers a novel ABA/auxin interaction in roots. Plant J. 28, 409–418. 11737778 10.1046/j.1365-313x.2001.01165.x

[tpj14596-bib-0091] Suzuki, M. , Wu, S. , Li, Q. and McCarty, D.R. (2014) Distinct functions of COAR and B3 domains of maize VP1 in induction of ectopic gene expression and plant developmental phenotypes in Arabidopsis. Plant Mol. Biol. 85, 179–191. 24473899 10.1007/s11103-014-0177-x

[tpj14596-bib-0092] Terrasson, E. , Buitink, J. , Righetti, K. ***et al*** (2013) An emerging picture of the seed desiccome: confirmed regulators and newcomers identified using transcriptome comparison. Front. Plant Sci. 4, 497. 24376450 10.3389/fpls.2013.00497 PMC3859232

[tpj14596-bib-0014] Van Buren, R. (2017) Desiccation tolerance: seedy origins of resurrection. Nat. Plants, 3, 17 046. 10.1038/nplants.2017.46 28346449

[tpj14596-bib-0015] Van Buren, R. , Bryant, D. , Edger, P.P. ***et al*** (2015) Single‐molecule sequencing of the desiccation‐tolerant grass *Oropetium thomaeum* . Nature, 527, 508–511. 26560029 10.1038/nature15714

[tpj14596-bib-0016] Van Buren, R. , Wai, C.M. , Pardo, J. , Giarola, V. , Ambrosini, S. , Song, X. and Bartels, D. (2018) Desiccation tolerance evolved through gene duplication and network rewiring in Lindernia. Plant Cell, 30, 2943–2958. 30361236 10.1105/tpc.18.00517 PMC6354263

[tpj14596-bib-0093] Vicente‐Carbajosa, J. and Carbonero, P. (2005) Seed maturation: developing an intrusive phase to accomplish a quiescent state. Int. J. Dev. Biol. 49, 645–651. 16096971 10.1387/ijdb.052046jc

[tpj14596-bib-0094] Wang, Guifeng , Wang, Gang , Zhang, X. , Wang, F. and Song, R. (2012) Isolation of high quality RNA from cereal seeds containing high levels of starch. Phytochem. Anal. 23, 159–163. 21739496 10.1002/pca.1337

[tpj14596-bib-0095] Wen, W. , Li, K. , Alseekh, S. ***et al*** (2015) Genetic determinants of the network of primary metabolism and their relationships to plant performance in a maize recombinant inbred line population. Plant Cell, 27, 1839–1856. 26187921 10.1105/tpc.15.00208 PMC4531352

[tpj14596-bib-0096] Xiao, L. , Yang, G. , Zhang, L. ***et al*** (2015) The resurrection genome of Boea hygrometrica: a blueprint for survival of dehydration. Proc. Natl. Acad. Sci. USA, 112, 5833–5837. 25902549 10.1073/pnas.1505811112 PMC4426394

[tpj14596-bib-0097] Yang, H. , Saitou, T. , Komeda, Y. , Harada, H. and Kamada, H. (1997) *Arabidopsis thaliana ECP63* encoding a LEA protein is located in chromosome 4. Gene, 184, 82–88. 9016956

[tpj14596-bib-0098] Yobi, A. , Schlauch, K.A. , Tillett, R.L. , Yim, W.C. , Espinoza, C. , Wone, B.W.M. , Cushman, J.C. and Oliver, M.J. (2017) *Sporobolus stapfianus*: insights into desiccation tolerance in the resurrection grasses from linking transcriptomics to metabolomics. BMC Plant Biol. 17, 67. 28351347 10.1186/s12870-017-1013-7 PMC5371216

[tpj14596-bib-0099] Yoo, S.‐D. , Cho, Y.‐H. and Sheen, J. (2007) Arabidopsis mesophyll protoplasts: a versatile cell system for transient gene expression analysis. Nat. Protoc. 2, 1565. 17585298 10.1038/nprot.2007.199

[tpj14596-bib-0100] Yoshida, T. , Fujita, Y. , Sayama, H. , Kidokoro, S. , Maruyama, K. , Mizoi, J. , Shinozaki, K. and Yamaguchi‐Shinozaki, K. (2010) AREB1, AREB2, and ABF3 are master transcription factors that cooperatively regulate ABRE‐dependent ABA signaling involved in drought stress tolerance and require ABA for full activation. Plant J. 61, 672–85. 19947981 10.1111/j.1365-313X.2009.04092.x

[tpj14596-bib-0101] Yoshida, T. , Fujita, Y. , Maruyama, K. , Mogami, J. , Todaka, D. , Shinozaki, K. and Yamaguchi‐Shinozaki, K. (2015) Four Arabidopsis AREB/ABF transcription factors function predominantly in gene expression downstream of SnRK2 kinases in abscisic acid signalling in response to osmotic stress. Plant. Cell Environ. 38, 35–49. 24738645 10.1111/pce.12351 PMC4302978

[tpj14596-bib-0102] Zdobnov, E.M. and Apweiler, R. (2001) InterProScan–an integration platform for the signature‐recognition methods in InterPro. Bioinformatics, 17, 847–848. 11590104 10.1093/bioinformatics/17.9.847

[tpj14596-bib-0103] Zhang, Q. and Bartels, D. (2018) Molecular responses to dehydration and desiccation in desiccation‐tolerant angiosperm plants. J. Exp. Bot. 69, 3211–3222. 29385548 10.1093/jxb/erx489

[tpj14596-bib-0104] Zheng, Y. , Jiao, C. , Sun, H. ***et al*** (2016) iTAK: a program for genome‐wide prediction and classification of plant transcription factors, transcriptional regulators, and protein kinases. Mol. Plant, 9, 1667–1670. 27717919 10.1016/j.molp.2016.09.014

