# Supplementary figures and images for: Vegetative desiccation tolerance in the resurrection plant Xerophyta humilis has not evolved through reactivation of the seed canonical LAFL regulatory network
Source: Plant J. 2019 Dec 10;101(6):1349–67. doi: 10.1111/tpj.14596 (PMC7187197; doi:10.1111/tpj.14596)

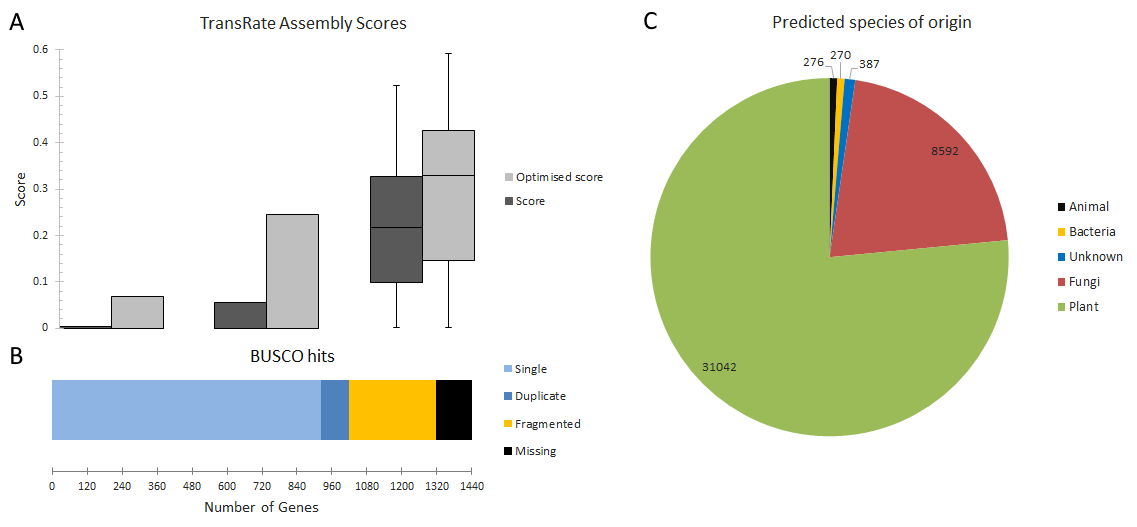

Supplement: Supplementary file 1 — Figure S1. Xerophyta humilis transcriptome assembly scores and taxonomic annotation. [file TPJ-101-1349-s001.tif]

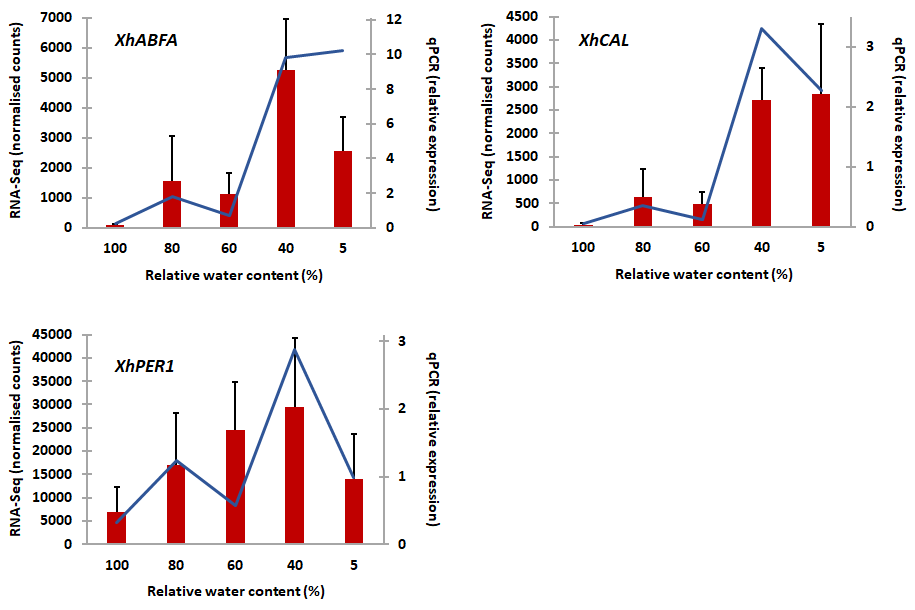

Supplement: Supplementary file 2 — Figure S2. Quantitative PCR analysis of mRNA levels of XhABFA, XhCAL and XhPER1 during desiccation of X. humilis. [file TPJ-101-1349-s002.tif]

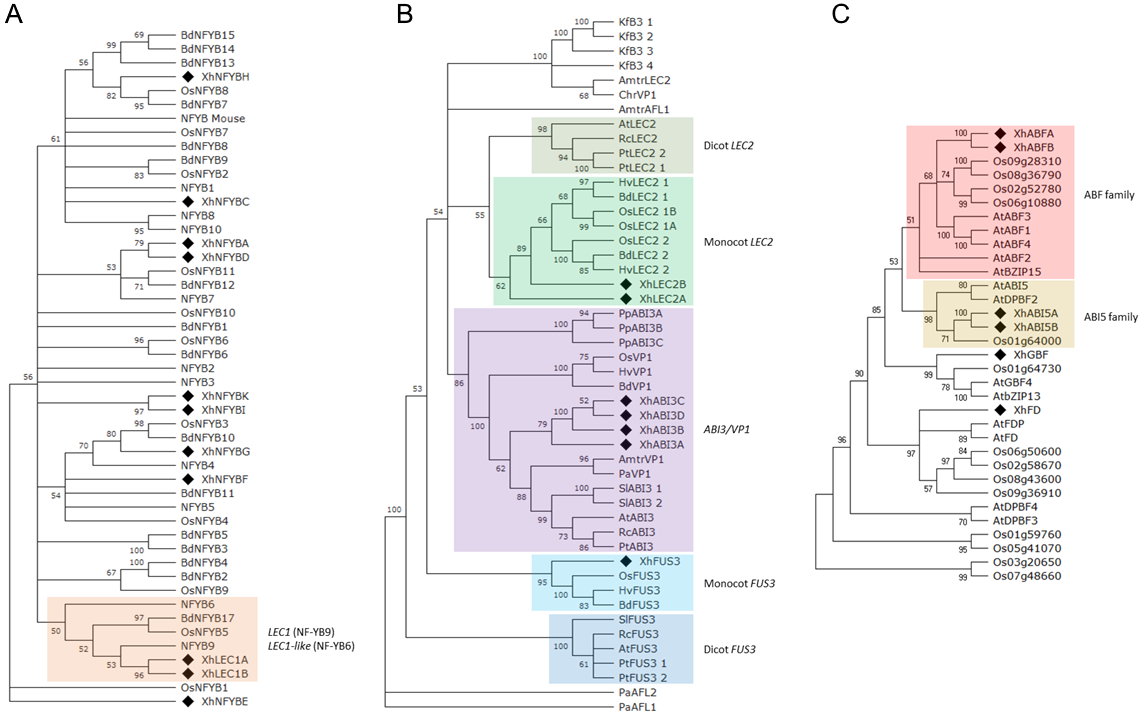

Supplement: Supplementary file 3 — Figure S3. Evolutionary relationship of LAFL genes across X. humilis and other species. [file TPJ-101-1349-s003.tif]

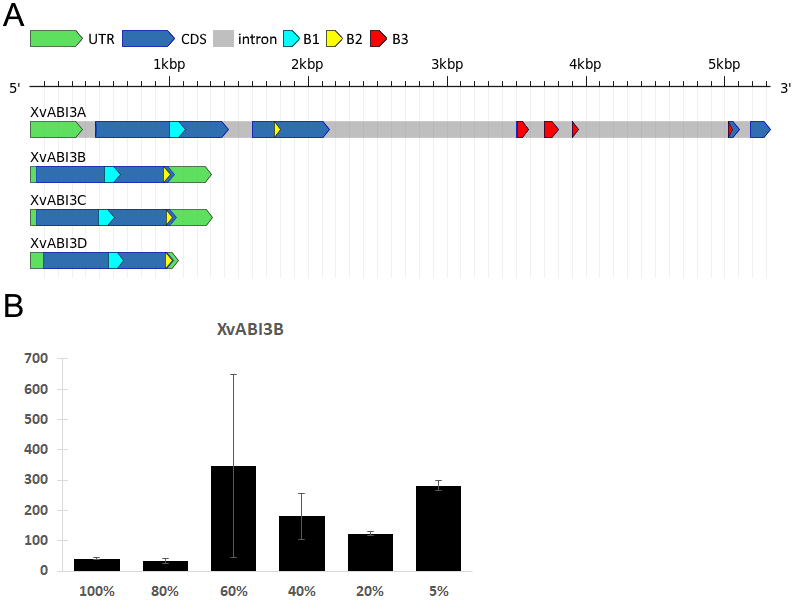

Supplement: Supplementary file 4 — Figure S4. Conservation of ABI3 paralogues in X. viscosa. [file TPJ-101-1349-s004.tif]

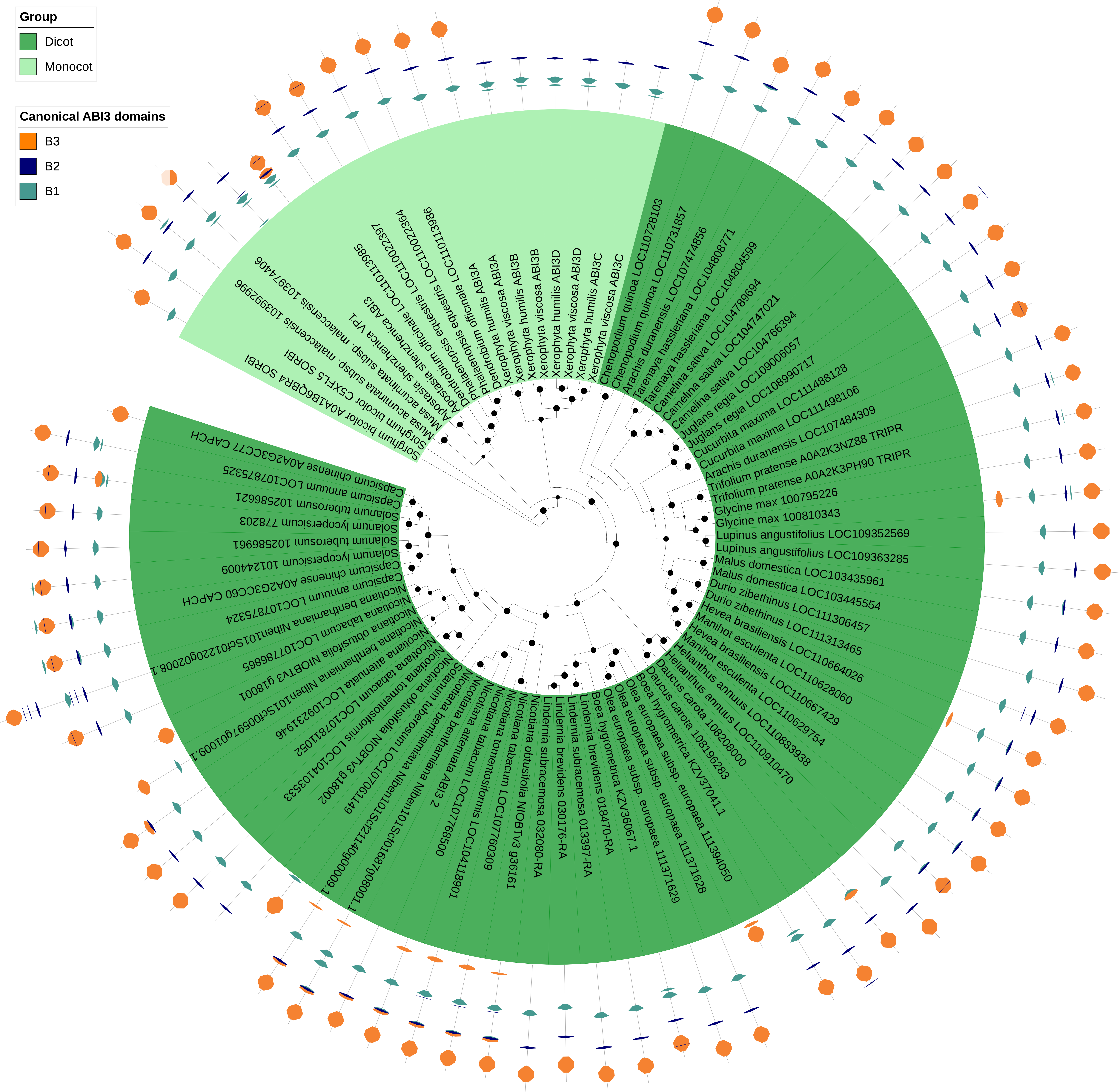

Supplement: Supplementary file 5 — Figure S5. Domain structure of ABI3 paralogues in angiosperms with duplicated ABI3. [file TPJ-101-1349-s005.png]

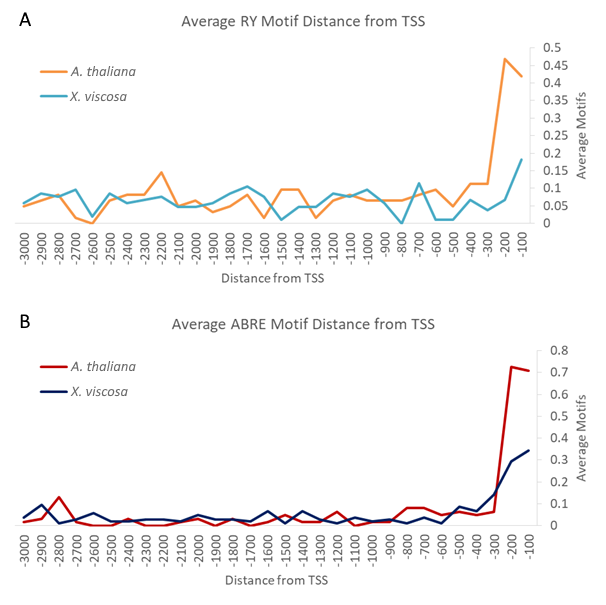

Supplement: Supplementary file 6 — Figure S6. Average motif distance from the TSS in A. thaliana and X. viscosa ABI3 regulon genes. [file TPJ-101-1349-s006.tif]

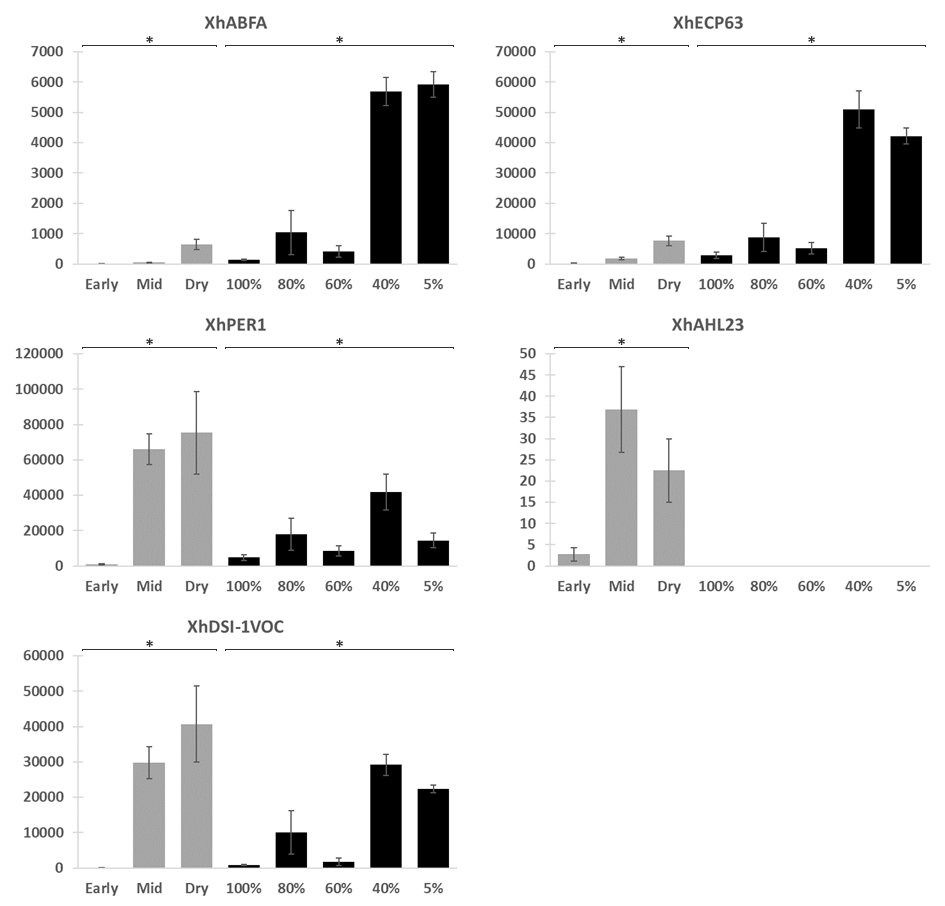

Supplement: Supplementary file 7 — Figure S7. Expression of XhABFA and putative target genes. [file TPJ-101-1349-s007.tif]

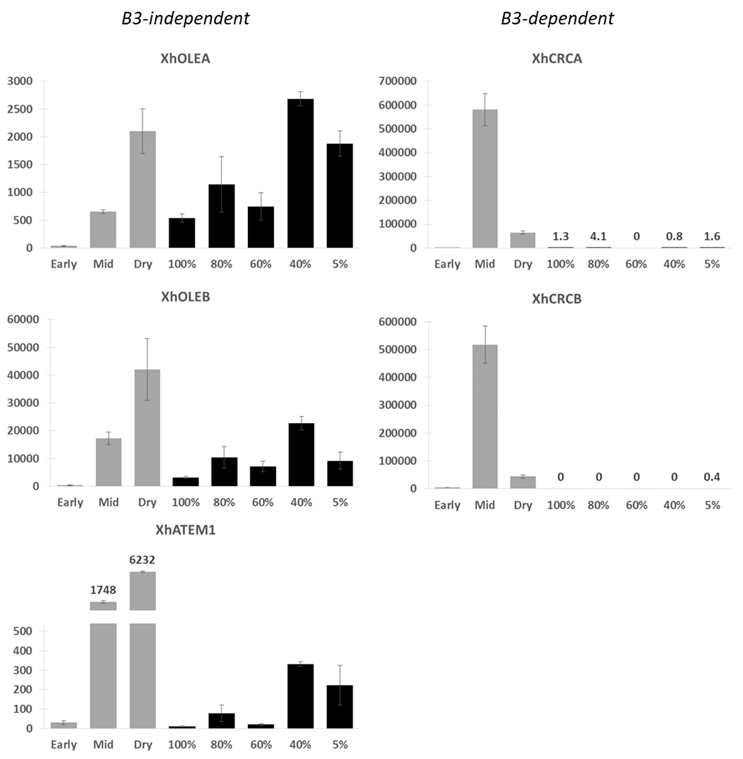

Supplement: Supplementary file 8 — Figure S8. Expression of several X. humilis seed genes. [file TPJ-101-1349-s008.tif]

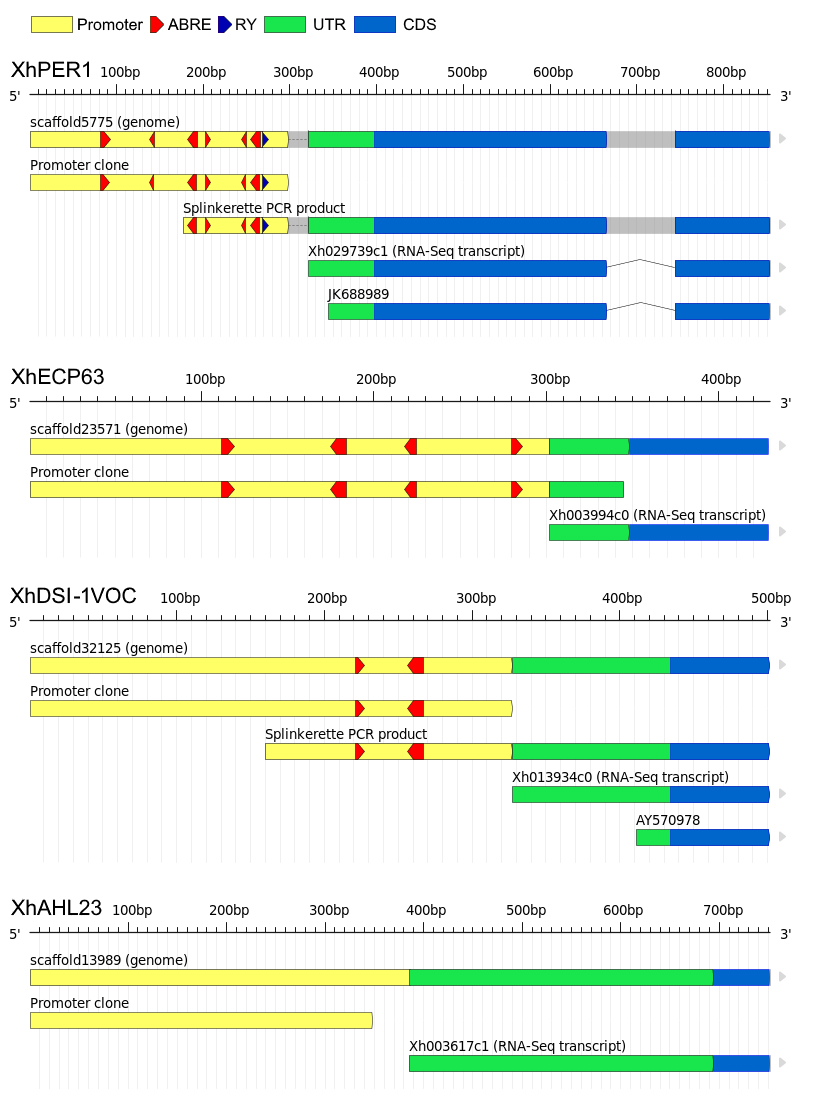

Supplement: Supplementary file 9 — Figure S9. Summary of promoter regions used in protoplast experiments. [file TPJ-101-1349-s009.tif]
