# Supplementary material for: Vegetative desiccation tolerance in the resurrection plant Xerophyta humilis has not evolved through reactivation of the seed canonical LAFL regulatory network
Source: Plant J. 2019 Dec 10;101(6):1349–67. doi: 10.1111/tpj.14596 (PMC7187197; doi:10.1111/tpj.14596)
Supplement: Supplementary file 10 — Table S1. Primers for quantitative PCR validation of RNA‐Seq data. [file TPJ-101-1349-s010.docx]

**Table S1. Primers for quantitative PCR validation of RNA-Seq data**

| Primer Name | Sequence 5’-3’ |
| --- | --- |
| ABFA-F | GCTCGGAAGCAGGTGAGTTA |
| ABFA-R | CCCATGCAAATGACAGATCCA |
| PER1 -F | TCTTGTTCTCCCACCCTGGA |
| PER1 -R | CGGCGTGTAGGCTTCGATAT |
| CAL1-F | CCTATGCAGGTTCAAGAGCA |
| CAL1-R | GCAGAGTTGCATAGCTTAAACC |
| MSRB5-F | CCGCGGGAGAGAATTCTATCG |
| MSRB5-R | CCTTCATGCGGAGGATACGAA |
