# Supplementary material for: Vegetative desiccation tolerance in the resurrection plant Xerophyta humilis has not evolved through reactivation of the seed canonical LAFL regulatory network
Source: Plant J. 2019 Dec 10;101(6):1349–67. doi: 10.1111/tpj.14596 (PMC7187197; doi:10.1111/tpj.14596)
Supplement: Supplementary file 11 — Table S2. PCR primers to amplify the XhABFA coding region, or promoter regions for XhPER1, XhECP63, XhDSI‐1VOC or XhAHL23 for cloning into vectors for plant protoplast experiments. [file TPJ-101-1349-s011.docx]

**Table S2. PCR primers to amplify the *XhABFA* coding region, or promoter regions for *XhPER1*, *XhECP63*, *XhDSI-1VOC* or *XhAHL23* for cloning into vectors for plant protoplast experiments.**

| Primer Name | Vector | Sequence 5’-3’ (restriction enzyme sites underlined) |
| --- | --- | --- |
| ABFAfor1 | pENTR | GCGCCGGTACCAAGATGAATTCGAAAACTAACATGC |
| ABFArev1 | pENTR | AAATATGCGGCCGCTGAATTTCAGTAACAGGAAGATG |
| Per1for1 | pENTR | GCGCCGGTACCAGAGAAAATAAACAGGATAAACCAC |
| Per1rev1 | pENTR | AAATATGCGGCCGCTGCTTAAGAAGGCGAGAGAG |
| ECP63for1 | pENTR | GCGCCGGTACCGACTTGTGCGTCGATATAGTATG |
| ECP63rev1 | pENTR | AAATATGCGGCCGCGATGAACTCTCGTAATCTGCTC |
| DSI-1VOCfor1 | pENTR | GCGCCGGTACCGAGCATAAAAATCAGCTCCTCAG |
| DSI-1VOCrev1 | pENTR | AAATATGCGGCCGCGAATCTAGCGAATAAAGTTGCAGC |
| AHL23for1 | pENTR | GCGCCGGTACCAGCTGCATGAATCTGTTGC |
| AHL23rev1 | pENTR | AAATATGCGGCCGCTAAAGGAGAGGTTGAGGAGC |
