# Supplementary material for: Vegetative desiccation tolerance in the resurrection plant Xerophyta humilis has not evolved through reactivation of the seed canonical LAFL regulatory network
Source: Plant J. 2019 Dec 10;101(6):1349–67. doi: 10.1111/tpj.14596 (PMC7187197; doi:10.1111/tpj.14596)
Supplement: Supplementary file 13 — Data S2. ABI3 3’‐RACE sequences. [file TPJ-101-1349-s013.rtf]

Supplemental Data S2. ABI3 3'-RACE sequences. Alignment of 3' RACE products to representative X. humilis A) ABI3A and B) ABI3B RNA-seq transcripts, showing the in planta expression of XhABI3B transcripts. The 3' RACE primers (yellow) amplified cDNA sequence for each respective gene, confirming the terminal position of the CDS (shown in colour) in each case and the presence of the early stop codon in ABI3B.
A)	XhABI3A
                       10        20        30        40        50        60        70        80        90       100                  
              ....|....|....|....|....|....|....|....|....|....|....|....|....|....|....|....|....|....|....|....|
XhABI3A       CATTTTGCCAAAGTGTTCTTAAAGCACTTCATTTCTTCCCAGCATTGCCAGGTGGCGGCGGCGGAACTGGCTGGCCGTGGCGGTGGGCCGCTGGTTTTTG 
XhABI3A RACE  ---------------------------------------------------------------------------------------------------- 

                      110       120       130       140       150       160       170       180       190       200         
              ....|....|....|....|....|....|....|....|....|....|....|....|....|....|....|....|....|....|....|....|
XhABI3A       TTGTTTCTGCGCACCAATCCTCCCTTGCTTACGCACAAGCTTTCCTTCTTCTCTGATCTCTACCTTCCCCAATCCTCATCGATCATCTTCATCTTCTCCT 
XhABI3A RACE  ---------------------------------------------------------------------------------------------------- 

                      210       220       230       240       250       260       270       280       290       300         
              ....|....|....|....|....|....|....|....|....|....|....|....|....|....|....|....|....|....|....|....|
XhABI3A       GAGAGATCTTTCTAGTTCGTCCATGAATTCTCAGTGCTCTTTGTAGATACTACATAAGTTATAGTAGAGAAGCTAAGCAGTCTCTATCTGATCTGTAGGT 
XhABI3A RACE  ---------------------------------------------------------------------------------------------------- 

                      310       320       330       340       350       360       370       380       390       400         
              ....|....|....|....|....|....|....|....|....|....|....|....|....|....|....|....|....|....|....|....|
XhABI3A       CTTCGTTTTCAGACCTTTCTGCGGGCCCTCCGTATCTCTCCATCTCTATCAACATCACCTTCTCTCAGAGAAGAACGATGGCGTCTGTCACTCGTGAAGA 
XhABI3A RACE  ---------------------------------------------------------------------------------------------------- 

                      410       420       430       440       450       460       470       480       490       500         
              ....|....|....|....|....|....|....|....|....|....|....|....|....|....|....|....|....|....|....|....|
XhABI3A       AAGAGACGGCGGAGGAGACGAGGTCATGGTGGAAATCGACGGCGAGGATTTCTTACTCTCCGACGAGCCGTTTCCTTCGCTCCCGGAATTTCCTTGTCTT 
XhABI3A RACE  ---------------------------------------------------------------------------------------------------- 

                      510       520       530       540       550       560       570       580       590       600         
              ....|....|....|....|....|....|....|....|....|....|....|....|....|....|....|....|....|....|....|....|
XhABI3A       CCTTCACCAGCGGCGTCACCGTCGCCGTCATCTACTTCAAACTACGCTAAAAGTAGCTCGTCAGCGTCATCATGGTCGTTCCTCAAAGTTCCTGGCGGTG 
XhABI3A RACE  ---------------------------------------------------------------------------------------------------- 

                      610       620       630       640       650       660       670       680       690       700         
              ....|....|....|....|....|....|....|....|....|....|....|....|....|....|....|....|....|....|....|....|
XhABI3A       CCGACGACATGCAGCAGGTTCCTGTCGTCCCTGACATCGTTACGGACGTCGGTCCTCCTCCGCCGCAGGATGCAGATCCGACAGCAAAGGAGAAATCGCA 
XhABI3A RACE  ---------------------------------------------------------------------------------------------------- 

                      710       720       730       740       750       760       770       780       790       800         
              ....|....|....|....|....|....|....|....|....|....|....|....|....|....|....|....|....|....|....|....|
XhABI3A       AGAAGAGTTCCAAGATATCCTAAGCAACTTGGACATTCTCAATCCATCTCTATTTGCCGACGATCAGACTGAGGCGTTGCCGGAGACGGATCAGAGTTCT 
XhABI3A RACE  ---------------------------------------------------------------------------------------------------- 

                      810       820       830       840       850       860       870       880       890       900         
              ....|....|....|....|....|....|....|....|....|....|....|....|....|....|....|....|....|....|....|....|
XhABI3A       TCGCATGTGTCGGCGGTGGGTGAAAACTGCTCCGACGATCTGGCGACTGTTTTCTTAAACTGGTTGAAAGACAACAAGGATTGCATTTCGCCGGAAGATC 
XhABI3A RACE  ---------------------------------------------------------------------------------------------------- 

                      910       920       930       940       950       960       970       980       990       1000        
              ....|....|....|....|....|....|....|....|....|....|....|....|....|....|....|....|....|....|....|....|
XhABI3A       TGAGGAGGATCAAACTCAAGAAGTCAACCGTCGAGTGCGCAGCCCGCCGCCTCGGCGGTGGGAATCAGGGGATGACGCAGCTGCTGAAGCTAATTTTAGA 
XhABI3A RACE  ---------------------------------------------------------------------------------------------------- 

                      1010      1020      1030      1040      1050      1060      1070      1080      1090      1100        
              ....|....|....|....|....|....|....|....|....|....|....|....|....|....|....|....|....|....|....|....|
XhABI3A       CTGGGTTCAGAACCACCACCTCCAGAGACGGCAGCATCATGGGGGAGACGTAGCAGAACAAGATCTACAAGAAGATGTCATACCTGAATTCTCCATTAAA 
XhABI3A RACE  ---------------------------------------------------------------------------------------------------- 


                      1110      1120      1130      1140      1150      1160      1170      1180      1190      1200        
              ....|....|....|....|....|....|....|....|....|....|....|....|....|....|....|....|....|....|....|....|
XhABI3A       GCTAATACTAATCTAGAGTATAAGAATAACTATGAGTCTGATAATAATATCATGTTATGCAATAGCTCCTGGATACCACAATCTTCTTCCTACACACAGG 
XhABI3A RACE  ---------------------------------------------------------------------------------------------------- 

                      1210      1220      1230      1240      1250      1260      1270      1280      1290      1300        
              ....|....|....|....|....|....|....|....|....|....|....|....|....|....|....|....|....|....|....|....|
XhABI3A       ATTCGGCGGCGGCGGGGCCTTCATTAATGATGACATACCCTATTGATCCCAGCTCTGCACCAGCATTTTCATTCGACGGGGCAGCCGGTGGTGTTACCGT 
XhABI3A RACE  -------------------------------------------------------------------------------------------------CGT 

                      1310      1320      1330      1340      1350      1360      1370      1380      1390      1400        
              ....|....|....|....|....|....|....|....|....|....|....|....|....|....|....|....|....|....|....|....|
XhABI3A       GACCACCCAGCCGTTTTCTGCAACAGATGAACTCCAACCCATGGACTCAGGGTCGTCTTGGCCGCATTTTTCTTCCCCGGCAGCACAATTTGCCGCCTAC 
XhABI3A RACE  GACCACCCAGCCGTTTTCTGCAACAGATGAACTCCAATCCATGGACTCAGGGTCATCTTGGCCGCATTTTCCTTCCCCGGCAGCACAATTTGTCGCCTAC 

                      1410      1420      1430      1440      1450      1460      1470      1480      1490      1500        
              ....|....|....|....|....|....|....|....|....|....|....|....|....|....|....|....|....|....|....|....|
XhABI3A       GCTAGGCCACAGCAGTATGCTGGTGGATATCCGGTGCAATATCCAGGGCAGTTGGTGGATCACCGACTCGCCAAAATAGCATCTGCAACGAGGGAGGCGA 
XhABI3A RACE  GACAGGCCACAGCAGTATGTTGGTGGATATCCGGTGCAATATCCAGGGCAGTTGGTGGATCACCGACTCGCCAAAATAGCATCTGCAACGAGGGAGGCGA 

                      1510      1520      1530      1540      1550      1560      1570      1580      1590      1600        
              ....|....|....|....|....|....|....|....|....|....|....|....|....|....|....|....|....|....|....|....|
XhABI3A       GGAAGAAGAGAATGGCAAGGCAGAGAAGGTTATCGTCTCTTCATCAACATCGGTATCAGAAGCAACACCGTGTTGATGAGTCTCGGTTTATATCTCAGTC 
XhABI3A RACE  GGAAGAAGAGAATGGCAAGGCAGAGACGGTTGTCGTCTCTTCATCAACATCGGAATCAGAAGCAACACCGTGTTGATGAGTCTCGGTTTATATCTCAGTC 

                      1610      1620      1630      1640      1650      1660      1670      1680      1690      1700        
              ....|....|....|....|....|....|....|....|....|....|....|....|....|....|....|....|....|....|....|....|
XhABI3A       CCAGCAACAACAGCAGCAACATTTCTGTTGTAGCAGAGATGGGGAAGGAGTGAATTACAGTCAGCAAAGTGGCTCTCGGAACTGCGCTTTCTGGTCTTCC 
XhABI3A RACE  CCAGCAACAACAGCAGCAACGTTTCTGTTGCAGTGGAGATGGGCAAGGAGTGAATTACAGTCAGCAAAGTAGCTCTAGGAACTGGGCTTTCTGGTCTTCC 

                      1710      1720      1730      1740      1750      1760      1770      1780      1790      1800        
              ....|....|....|....|....|....|....|....|....|....|....|....|....|....|....|....|....|....|....|....|
XhABI3A       ATGGCTTCATTGCCCTCACAACAGATGCATTCTCTTCTAGAAAGTTCTAATCCGTCGCTGGATCCTCGCAACATAGAACCACCATCACCGCGGTCATCCA 
XhABI3A RACE  ATGGCTTCATTGCCCTCACAACAGATGCATTCTCTTCTAGAAGGTTCTAATCCGTCGCCGGATCCTCGCAACACAGAACCACCATCACCGCGTTCATCCC 

                      1810      1820      1830      1840      1850      1860      1870      1880      1890      1900        
              ....|....|....|....|....|....|....|....|....|....|....|....|....|....|....|....|....|....|....|....|
XhABI3A       CCATACCGCATAGCATTCAGCAGTATTCAAACCCCTCGGAGCGGCGGCAGGGATGGAAAGTCGAGAAGAACTTGAAGTTTCTCCTACAAAAGGTGCTGAA 
XhABI3A RACE  CCATACCACATAGCATTCAGCACTATTCAAACCCGTTGGAGCGGCGGCAGGGATGGAAAGTCGAGAAGAACTTGAAGTTTCTCCTACAAAAGGTGCTGAA 

                      1910      1920      1930      1940      1950      1960      1970      1980      1990      2000        
              ....|....|....|....|....|....|....|....|....|....|....|....|....|....|....|....|....|....|....|....|
XhABI3A       GCAGAGCGATGTTGGTAGCCTCGGAAGGATTGTGCTGCCTAAAAAAGAGGCAGAGATTCATCTTCCTGAACTGACTGCAAGGGATGGAATCTCCATACCT 
XhABI3A RACE  GCAGAGCGATGTTGGTAGCCTCGGAAGGATCGTGCTGCCTAAAAAAGAGGCAGAGATTCATCTTCCTGAACTGACTGCAAGGGATGGAATCTCCATACCT 

                      2010      2020      2030      2040      2050      2060      2070      2080      2090      2100        
              ....|....|....|....|....|....|....|....|....|....|....|....|....|....|....|....|....|....|....|....|
XhABI3A       ATGGAGGATATTGGAACTTCAAGAGTATGGAACATGAGATACAGGTTTTGGCCCAACAACAAGAGCAGAATGTATCTTTTGGAAAATACAGGAGACTTTG 
XhABI3A RACE  ATGGAGGATATTGGAACTTCAAGAGTATGGAACATGAGATACAGGTTTTGGCCCAATAACAAGAGCAGAATGTATCTTTTGGAAAATACAGGAGACTTTG 

                      2110      2120      2130      2140      2150      2160      2170      2180      2190      2200        
              ....|....|....|....|....|....|....|....|....|....|....|....|....|....|....|....|....|....|....|....|
XhABI3A       TGAGATGCAATGCGCTTCAAGAAGGAGACTTCATAGTCATCTACTCGGATGTTAAACGAGGAAAATATATGATAAGAGGAGTAAAGGTACGTCAGCCCAC 
XhABI3A RACE  TGAGATGCAATGCGCTTCAAGAAGGAGACTTCATAGTCATCTACTCGGATGTTAAACGCGGAAAATATATGATAAGAGGAGTAAAGGTACGTCAGCCCAC 


                      2210      2220      2230      2240      2250      2260      2270      2280      2290      2300        
              ....|....|....|....|....|....|....|....|....|....|....|....|....|....|....|....|....|....|....|....|
XhABI3A       AGAACTCAAAGCTATCGGCAACAAGAATGCGAGACAGAAACAGATCGGAGCAGAAAATCGAAGCTTACAGAAGTCGAAGAACACCGACAAAAGCTCAAGG 
XhABI3A RACE  AGAACTCAAAGCTATCGGCAGCAAGAATGCGAGACAGAAACAGATCGGAGCAGAAAATCGAAGCTTGCAGAAGTCGATGACCMCCGACAAAARCYCAAGG 

                      2310      2320      2330      2340      2350      2360      2370      2380      2390      2400        
              ....|....|....|....|....|....|....|....|....|....|....|....|....|....|....|....|....|....|....|....|
XhABI3A       AATTTGGATAGTAAGATGAAAAAACTCCCATCTGCTGCTAAAACTGATACCAACTATGAGCAAAGAAATTGAAAA-CAAATATAATTTCCGAAGTTTTGG 
XhABI3A RACE  AATTTGGAWAGTAARATGAAAAAACYCCCATYTGCTGCTAAAACTGATMCCAACTATGAGCAAARAAATTGAAAAACAAAWAWAATTTCCAAAGTTTTGG 

                      2410      2420      2430      2440      2450      2460      2470      2480      2490      2500        
              ....|....|....|....|....|....|....|....|....|....|....|....|....|....|....|....|....|....|....|....|
XhABI3A       TTTGTTCACTTGTATGCGAAAAATGTTACTACTACTATAAACACCATATATGCGCGAAACTTATGTACAATATGAGTTTTTATTTTAATTCTGATAAAAA 
XhABI3A RACE  TTTGTTCACTTGTATGCGAAAAATGTTACTATTAAAAAAAAAAAAAAAAAAAAAAAAAAAAAAAAAAAA------------------------------- 

                      2510      2520      2530      2540      2550      2560      2570      2580      2590      2600        
              ....|....|....|....|....|....|....|....|....|....|....|....|....|....|....|....|....|....|....|....|
XhABI3A       TTGTCGGTTCACGTACACATTCAAATTGTCGGGTTTTTTTAGCCAATTGGATGAATTACATGTCCATTTCCGGTACGTGAGCCTTTGTGGTACCGGATAT 
XhABI3A RACE  ---------------------------------------------------------------------------------------------------- 

                      2610      2620      2630      2640      2650      2660      2670      2680      2690      2700        
              ....|....|....|....|....|....|....|....|....|....|....|....|....|....|....|....|....|....|....|....|
XhABI3A       TCTTTTTTTTTAACGACGGACAATGTTGTGAACTGTATTTCTCCCTCAC--------------------------------------------------- 
XhABI3A RACE  ---------------------------------------------------------------------------------------------------- 

                      2710      2720      2730      2740      2750      2760      2770      2780      2790      2800        
              ....|....|....|....|....|....|....|....|....|....|....|....|....|....|....|....|....|....|....|....|
XhABI3A       ---------------------------------------------------------------------------------------------------- 
XhABI3A RACE  ---------------------------------------------------------------------------------------------------- 

                      2810      2820      2830      2840      2850      2860          
              ....|....|....|....|....|....|....|....|....|....|....|....|....|..
XhABI3A       ------------------------------------------------------------------- 
XhABI3A RACE  ------------------------------------------------------------------- 
B)	
XhABI3B

                       10        20        30        40        50        60        70        80        90       100                  
              ....|....|....|....|....|....|....|....|....|....|....|....|....|....|....|....|....|....|....|....|
XhABI3B       CAATCAATAAGTAAGCTACCGCAAAAGACATTATCGATGCTCGATTATGCAAAATTCGGAGAGAATATTTCGGATGGCAAATCGGACAAGTTATTCGAAG 
XhABI3B RACE  ---------------------------------------------------------------------------------------------------- 

                      110       120       130       140       150       160       170       180       190       200         
              ....|....|....|....|....|....|....|....|....|....|....|....|....|....|....|....|....|....|....|....|
XhABI3B       AACAGACCTAGGTTACCTTATTAGGCAGTATTCGCTATAAACAGTTCCATAGCTGGCAAGTTGGGCGGGGAAGAAGATAGAAGCAAGCCGAGGTCCCTGT 
XhABI3B RACE  ---------------------------------------------------------------------------------------------------- 

                      210       220       230       240       250       260       270       280       290       300         
              ....|....|....|....|....|....|....|....|....|....|....|....|....|....|....|....|....|....|....|....|
XhABI3B       CGCTGCTTCCCAGTTCCCGGCAATCTTCTCTTCCTAATCACACACCGTGCTATCCTCTCAATAGTCCTAGTCATCCTCTTATTTCAAAGAGAGATGGTGA 
XhABI3B RACE  ---------------------------------------------------------------------------------------------------- 

                      310       320       330       340       350       360       370       380       390       400         
              ....|....|....|....|....|....|....|....|....|....|....|....|....|....|....|....|....|....|....|....|
XhABI3B       ACACTGGTAATTCATGGGAGGAAGAAAGAGGTGATGACGTCGTGGTAGATATAGACGGTGAGGATTGCATCTTCTCCATTGATCTCATCGCACCATCGCT 
XhABI3B RACE  ---------------------------------------------------------------------------------------------------- 

                      410       420       430       440       450       460       470       480       490       500         
              ....|....|....|....|....|....|....|....|....|....|....|....|....|....|....|....|....|....|....|....|
XhABI3B       TCCAGATTTTTCCTCCCCCACATCCCCGTCGCACTCCTCGCCATCGCCGTCGTTGGCTTCCTCGAAGTTGAAGTCGGCGGATGCGGGTGCGCTGCCGCCG 
XhABI3B RACE  ---------------------------------------------------------------------------------------------------- 

                      510       520       530       540       550       560       570       580       590       600         
              ....|....|....|....|....|....|....|....|....|....|....|....|....|....|....|....|....|....|....|....|
XhABI3B       CCGCCGGCTGCGGAGGATTCCGCCCAGTCGATGTGGAATCTCGTCGCCGATATCGATCTGCTCGATCCTTGTAATCCTTTTTATGACGTCTATACGTCGA 
XhABI3B RACE  ---------------------------------------------------------------------------------------------------- 

                      610       620       630       640       650       660       670       680       690       700         
              ....|....|....|....|....|....|....|....|....|....|....|....|....|....|....|....|....|....|....|....|
XhABI3B       TAGATGAAACCAACATTAGCGCCCTGGTGTCTGACAAAGACATCTCCGTCGATGAGCATCAGCTCGTCGGCGGCGGCGTGGGGGAGATCTCGTTGAACTC 
XhABI3B RACE  ---------------------------------------------------------------------------------------------------- 

                      710       720       730       740       750       760       770       780       790       800         
              ....|....|....|....|....|....|....|....|....|....|....|....|....|....|....|....|....|....|....|....|
XhABI3B       GTCGGATGATCTGGGGAAGCTATTTTTGGAGTGGCTTAAGGACAACAAAGATGCGATTTCACCTGAAGATCTGAGGAGCATTAAGCTCAAGCGATCGACA 
XhABI3B RACE  ---------------------------------------------------------------------------------------------------- 

                      810       820       830       840       850       860       870       880       890       900         
              ....|....|....|....|....|....|....|....|....|....|....|....|....|....|....|....|....|....|....|....|
XhABI3B       ATCGAGTGTGCTGTACGCCGCCTGGGCGGTGGAAGCGAAGGTATGAAGAAGCTCCTGAAGCTCATCCTACATTGGGTTCGGAATAACCATTTACATAAGA 
XhABI3B RACE  ---------------------------------------------------------------------------------------------------- 

                      910       920       930       940       950       960       970       980       990       1000        
              ....|....|....|....|....|....|....|....|....|....|....|....|....|....|....|....|....|....|....|....|
XhABI3B       AAAAACAGCAACAGCAGCAGCAGCAGCAACAACATCAACTATATGCACCTTTTGCTTTCCAGCAGCCGGATTTCTCAAATTCAGTAACTGTAAGTTCCCC 
XhABI3B RACE  ---------------------------------------------------------------------------------------------------- 

                      1010      1020      1030      1040      1050      1060      1070      1080      1090      1100        
              ....|....|....|....|....|....|....|....|....|....|....|....|....|....|....|....|....|....|....|....|
XhABI3B       TGTATCGCCATATTGCAGTAGTGCAAGAAATGGAGCTTTTATGCAATTTTCTCCGCTGCTGGACCAGCAGAATGTTGAAAATCAAAATTCTTGGCCGTCA 
XhABI3B RACE  ----------------------------------------------------------------------------------------------CCGTC 


                      1110      1120      1130      1140      1150      1160      1170      1180      1190      1200        
              ....|....|....|....|....|....|....|....|....|....|....|....|....|....|....|....|....|....|....|....|
XhABI3B       TCCCCTGCTCCAAGTCCAAGACAGTATGGATCCTACAGTAGATCCTCTTTTGGGAATTCGCTAACTCCACAGCAAAATGGAATGGCGCCAGGCCGCTGTG 
XhABI3B RACE  TCCCCTGCTCCAAGTCCAAGACAATATGGATCCTACAGTAGATCCTCTTTTGGGAATTCGCTAACTCCACTGCAAAATGGAATGACGCCAGGCCGCTGTG 

                      1210      1220      1230      1240      1250      1260      1270      1280      1290      1300        
              ....|....|....|....|....|....|....|....|....|....|....|....|....|....|....|....|....|....|....|....|
XhABI3B       AGGCCAAGGAAGCGCGTAAGAAGAGAATGGCAAGGCACAGACGCTTCTCTTCTATCAACGCTAATTGCATTTAGAGGAACTGTTTGTAACTAGTTATCAT 
XhABI3B RACE  AGGCCAAGGAAGCTCGTAAGAAGAGAATGGCAAGGCACAGACGCTTCTCTTCTATCAACGCTAATTGCATTTAGAGGAACTATTTGTAACTAGTTATCGT 

                      1310      1320      1330      1340      1350      1360      1370      1380      1390      1400        
              ....|....|....|....|....|....|....|....|....|....|....|....|....|....|....|....|....|....|....|....|
XhABI3B       AAGTAACTTTGTCACGGCAACTTGTTCAATATCATAAGTTGTCTAATTGATTCCACGTCCTGTAAGATGACTTCCCTTATGGCACAATATTGTAGGTTTC 
XhABI3B RACE  AAGTAACTTTGTCACGGCAACTTGTTCAATATCATAAGTTGTCTAATTGATTCCACGTCCTGTAAGATGACTTCCGTTATGGTACAATATTGTAGGTTTC 

                      1410      1420      1430      1440      1450      1460      1470      1480      1490      1500        
              ....|....|....|....|....|....|....|....|....|....|....|....|....|....|....|....|....|....|....|....|
XhABI3B       TTTCAGTAGTTATGATCTTATAAGGACCTATGTATTAATTTTGATAATATAGCAAAATGAATGAATAGCTTTAGTGCTGTACTGCATCTGTAGTTGCAGC 
XhABI3B RACE  TTGCAGTAGTTATGATCTTATAAGGACCTGTGTATTAATTTTGAAAATATAGCAAAATGAATGAATAGCTTCAGTGCTGTACTGCATCTGTAGTTGCAGG 

                      1510      1520      1530      1540      1550      1560      1570      1580      1590      1600        
              ....|....|....|....|....|....|....|....|....|....|....|....|....|....|....|....|....|....|....|....|
XhABI3B       TGAACAAGATTCCTTGCTGAACAAGATTCCTTCTCTAGAAAACGTAGTCTCTTGCTAAAGTTGCTGATTTTCTCTATGTAAGGAAGCTTTTCTTCGAAGT 
XhABI3B RACE  TGAAC-----------------AAGATTTCTTCTCTAGAAAACATAGTCTCTTGCTAAAGTTGCTGATTTTCTCTATGTAAGGAAGCTTTTCTTGGAAGT 

                      1610      1620      1630      1640      1650      1660      1670      1680      1690      1700        
              ....|....|....|....|....|....|....|....|....|....|....|....|....|....|....|....|....|....|....|....|
XhABI3B       AGGTTGAAGGTTAAGCCACAATTGTTCATACCATCTTCTAGTCGTCTTAACCACGTTATAATGGAGAATTGAGATGATTACCATATATTATAACAACTGT 
XhABI3B RACE  AGTTTGAAGGTTAAGCCACAATTGTTCATACCATCTTCTAGTCGTCTTGACCACGTGACAATGGAGAATTAAGATGATTACCATATATTATA---ACTGT 

                      1710      1720      1730      1740      1750      1760      1770      1780      1790      1800        
              ....|....|....|....|....|....|....|....|....|....|....|....|....|....|....|....|....|....|....|....|
XhABI3B       AGCTACAGTGGAATATGGGTCATATTTGCGTTT-ATCTATTGAATTAGGCAATTGGGTGGGGGCAAAATAGAATGCCTCCAGCTTTCTTCAGTTGCAAGA 
XhABI3B RACE  AGCTACAGTGGAATATGG-TCATATTTGCGTTTTATCTATTGAATTAGGTAATTGAGTGGGGGCAAACCAGAATGCCTCCAGCTTTCTTCAGTTGCAAGA 

                      1810      1820      1830      1840      1850      1860      1870      1880      1890      1900        
              ....|....|....|....|....|....|....|....|....|....|....|....|....|....|....|....|....|....|....|....|
XhABI3B       TATGTTGCTGTTAAAAGGATGAGATAGTTTGGAAGTTTTGTTTCCACTGTTATTAGCAGAGTGGCAGGTATCCTTTTATCTGGTTACTATCATTTCTTCC 
XhABI3B RACE  TATATTGCAGTTAAAAGGATGAGATAGTTTGGAAGTTTTGTT-CCMCTGTTATTAGCAGAGKGGCAGGTATCCTTTTATCKGGTTACTATCATTTCTTCC 

                      1910      1920      1930      1940      1950      1960      1970      1980      1990      2000        
              ....|....|....|....|....|....|....|....|....|....|....|....|....|....|....|....|....|....|....|....|
XhABI3B       TACTCACCCTTTCTATGGTGATTGGTGAAGCTGGTGGTTGTAACTGCATCAGCAGCTCCAAGCGGTATCCCTACTTGTGTTTATCCTTGTTTCTATTTTT 
XhABI3B RACE  TATTCMCCCTTTYTATGGKGATTGGKGAAGCTGGKGGTKGTAACTGCATCAGCAGCTCCAAGCGGTATCCCTACTTGKGTTTATCCTKGTATYTATTTTT

                      2010      2020      2030      2040      2050      2060      2070      2080      2090      2100        
              ....|....|....|....|....|....|....|....|....|....|....|....|....|....|....|....|....|....|....|....|
XhABI3B       GTACTCCATAGTTACTCTTTGGTGTGATTTTCAAAGACTTTTGGTTTGTAAATTGTCATACTTTGTGTTCGTCTATCGCATCAGATTCAACTGGATTTAT 
XhABI3B RACE  TTACTCSGTAGTTACTYTTTGGKGTGATCTTTTTAGACTTTTGGTTTGTAAATTGTTATACTTKGTGTTCGTCGATCMCATCAGATTAAACKGGTTTTWT 

                      2110      2120      2130      2140      2150      2160      2170      2180      2190      2200        
              ....|....|....|....|....|....|....|....|....|....|....|....|....|....|....|....|....|....|....|....|
XhABI3B       TTGCTTCATTGACATTTGGTATCGTTCACTTTGACTTTGTCTTTCTCATTTTCATTGTTCTTAGTAAGCTGCTGAAACCTGTTATTCCAGATGACTTTGC 
XhABI3B RACE  TTGCTTCAAAAAAAAAAAAAAAAAAAAAAAAAAAAA---------------------------------------------------------------- 


                      2210      2220      2230      2240      2250      2260      2270      2280      2290      2300        
              ....|....|....|....|....|....|....|....|....|....|....|....|....|....|....|....|....|....|....|....|
XhABI3B       ATCTTTGGAGAGACACATCTTTTGATACACCAGGGTTGTTCCTGCCTTTTGCTGTTAATCATTTAGAACAGTCATTTCTGAGTCGAACACGAGTATCCAG 
XhABI3B RACE  ---------------------------------------------------------------------------------------------------- 

                      2310      2320      2330      2340      2350      2360      2370      2380      2390      2400        
              ....|....|....|....|....|....|....|....|....|....|....|....|....|....|....|....|....|....|....|....|
XhABI3B       GGGGTTTGAGGTTGATAATTATCCCTACTGACTGATGAGGTATATCCCATGTTACATTCAAAATGGGATCCAGTGACCAGAAACACTTCATTTTATGCTT 
XhABI3B RACE  ---------------------------------------------------------------------------------------------------- 

                      2410      2420      2430      2440      2450      2460      2470      2480      2490      2500        
              ....|....|....|....|....|....|....|....|....|....|....|....|....|....|....|....|....|....|....|....|
XhABI3B       GCTCCATTGACTTTTACGAGCATCGTATGCGATTATCAAGGTTTACAGCTTCTTGATTTAGATCGTTATCCTGCATAGGCTTTCAGCAAGCGGGATGCTT 
XhABI3B RACE  ---------------------------------------------------------------------------------------------------- 

                      2510      2520      2530      2540      2550      2560      2570      2580      2590      2600        
              ....|....|....|....|....|....|....|....|....|....|....|....|....|....|....|....|....|....|....|....|
XhABI3B       GTGGGATTTGCTTGGTTTGATTTTTAATGAGTTGGTTATTCTTGTAAAGTTTCTTGCAGATTTGAATACATACAAATCTTCAAAATTCACATAGCATGTG 
XhABI3B RACE  ---------------------------------------------------------------------------------------------------- 

                      2610      2620      2630      2640      2650      2660      2670      2680      2690      2700        
              ....|....|....|....|....|....|....|....|....|....|....|....|....|....|....|....|....|....|....|....|
XhABI3B       TTTCTTTACAAATGATTGAGCTCGAGACTACACCGATCTTTTCTTGATGTTGATTATCTGGAGTTGATAGCTGATCTGATATGATGCTTCTCTAAGATGT 
XhABI3B RACE  ---------------------------------------------------------------------------------------------------- 

                      2710      2720      2730      2740      2750      2760      2770      2780      2790      2800        
              ....|....|....|....|....|....|....|....|....|....|....|....|....|....|....|....|....|....|....|....|
XhABI3B       CATTGGTTGCTTGAGGCTAACTTACCAAGCTTTAGTGAATGTCTTGTAGAGTCCGCTTTGGTATTCAAATGTTCAGGTTTATGTTTTACTGAAATGTGTT 
XhABI3B RACE  ---------------------------------------------------------------------------------------------------- 

                      2810      2820      2830      2840      2850      2860          
              ....|....|....|....|....|....|....|....|....|....|....|....|....|..
XhABI3B       ACTCGCAAGTTTGATTATCCATTTGTTATACTTCGGATTTTGGGAAATAAAGAAAATTTGGCTGATA 
XhABI3B RACE  ------------------------------------------------------------------- 
