# Supplementary material for: Vegetative desiccation tolerance in the resurrection plant Xerophyta humilis has not evolved through reactivation of the seed canonical LAFL regulatory network
Source: Plant J. 2019 Dec 10;101(6):1349–67. doi: 10.1111/tpj.14596 (PMC7187197; doi:10.1111/tpj.14596)
Supplement: Supplementary file 14 [file TPJ-101-1349-s014.docx]

**Supplementary Figures, Tables and Data**

**Figure S1. *X. humilis* transcriptome assembly scores and taxonomic annotation.** A) TransRate score (black bars) and optimised score (grey bars) for the raw and RapClust *X. humilis* assemblies, compared to the scores calculated for 155 published transcriptomes (Smith-Unna, 2015). B) Completeness of the RapClust representative *X. humilis* transcriptome as measured against the BUSCO gene set. C) Distribution of taxonomic origin of transcripts with a hit in the Swissprot and UniRef90 databases.

**Figure S2. Quantitative PCR analysis of mRNA levels of *XhABFA*, *XhCAL* and *XhPER1* during desiccation of *X. humilis***. RNA-Seq expression values (blue lines) are normalised read counts. Relative expression was determined by qPCR (red bars) by normalisation to the expression level of *XhMSRB5*. Values shown are means +SEM for three biological repeats.

**Figure S3. Evolutionary relationship of LAFL genes across *X. humilis* and other species.** Maximum-likelihood phylogenies of (A) NF-YB genes (B) AFL genes and (C) group-A bZIPs across several species, calculated with 1000 bootstrap iterations. Sequence data based on that collected by Petroni *et al.*, 2012 (NF-YB) and Carbonero *et al.,* 2016 (AFL). Multiple alignments created using Clustal Omega, and phylogenetic trees created using RAxML.

**Figure S4. Conservation of ABI3 paralogues in *X. viscosa*.** A) Multiple exon organisation of *XvABI3A*, encoding full-length ABI3 protein, compared to the truncated *XvABI3* paralogues found on only a single exon. B) DESeq2 normalised counts of *XvABI3B* in desiccating *X. viscosa* leaves.

**Figure S5: Domain structure of ABI3 paralogues in angiosperms with duplicated ABI3.** Additional ABI3 protein sequences were obtained from OrthoDB and aligned using Clustal Omega. Custom HMMs were used to find the coordinates of the B1, B2 and B3 domains in all proteins using PFAM hmmscan (teal, blue and orange, respectively). A maximum-likelihood phylogeny was calculated using FastTree and visualised using EMBL Interactive Tree of Life. There is clear separation of the monocot and eudicot clades (light and dark green).

**Figure S6. Average motif distance from the TSS in *A. thaliana* and *X. viscosa* ABI3 regulon genes.** Average number of (A) RY and (B) ABRE motifs found up to 3000 bp upstream of the corresponding gene TSS. Both motifs are predominantly found within 300 bp of the TSS in *A. thaliana*, whereas only the ABRE is enriched in *X. viscosa* promoters.

**Figure S7. Expression of *XhABFA* and putative target genes.** DESeq2 normalised counts of the *X. humilis* bZIP *XhABFA*, the seed-specific genes *XhDSI1-VOC* and 1-Cys peroxiredoxin *XhPER1*, the LEA *XhECP63*, and control gene *XhAHL23* during seed maturation (grey) and VDT (black). Counts from both tissues are displayed on the same axis but are derived from different experiments. Error bars are standard error. (*) indicates significant at FDR < 0.01.

**Figure S8**: **Expression of several *X. humilis* seed genes.** DESeq2 normalised counts of *X. humilis* orthologues of B3-independent *A. thaliana* genes (*OLE2*, *At2g25890* and *AtEM1*) and B3-dependent genes (*AtCRC)* during seed maturation (grey) and VDT (black) as observed by Suzuki et al. 2014. Counts from both tissues are displayed on the same axis but are derived from different experiments. Error bars are standard error.

**Figure S9. Summary of promoter regions used in protoplast experiments.** Promoters for *XhPE*R1, *XhECP63*, *XhDSI-1VOC* and *XhAHL23* were aligned to the *X. humilis* genome assembly scaffolds, and their positions relative to start sites for RNA-Seq transcripts are summarized. The results of Splinkerette PCR experiments and independent cDNA clones for *XhPER1* and *XhDSI-1VOC*, validating the integrity of these assemblies are included. Conserved ABRE and RY cis-regulatory elements in these promoters are indicated.

**Table S1. Primers for quantitative PCR validation of RNA-Seq data**

**Table S2. PCR primers to amplify the *XhABFA* coding region, or promoter regions for *XhPER1*, *XhECP63*, *XhDSI-1VOC* or *XhAHL23* for cloning into vectors for plant protoplast experiments.**

**Data S1: Expression data for genes analysed in Figure 4.** Homologues of *A. thaliana* seed genes identified in *X. humilis* used to create the heatmaps in Fig. 4 including DESeq2 normalised counts across the analysed sample conditions. Average expression data and GO terms of homologues of *A. thaliana* seed genes, which were not differentially expressed in either *Xerophyta* seed development or leaf desiccation are also given.

**Data S2. *ABI3* 3’-RACE sequences.** Alignment of 3’ RACE products to representative *X. humilis* A) *ABI3A* and B) *ABI3B* RNA-seq transcripts, showing the *in planta* expression of *XhABI3B* transcripts. The 3’ RACE primers (yellow) amplified cDNA sequence for each respective gene, confirming the terminal position of the CDS (shown in colour) in each case and the presence of the early stop codon in *ABI3B*.
